# Selectivity analyses of γ-benzylidene digoxin derivatives to different Na,K-ATPase α isoforms: a molecular docking approach

**DOI:** 10.1080/14756366.2017.1380637

**Published:** 2017-11-08

**Authors:** Marco T. C. Pessôa, Silmara L. G. Alves, Alex G. Taranto, José A. F. P. Villar, Gustavo Blanco, Leandro A. Barbosa

**Affiliations:** a Laboratório de Bioquímica Celular, Universidade Federal de São João del Rei, Campus Centro-Oeste Dona Lindú, Divinópolis, Brazil;; b Laboratório de Síntese Orgânica e Nanoestruturas, Universidade Federal de São João del Rei, Campus Centro-Oeste Dona Lindú, Divinópolis, Brazil;; c Laboratório de Modelagem Molecular, Universidade Federal de São João del Rei, Campus Centro-Oeste Dona Lindú, Divinópolis, Brazil;; d Department of Molecular and Integrative Physiology, Kansas University Medical Center, Kansas City, KS, USA

**Keywords:** Cardiotonic steroids, digoxin, Na,K-ATPase isoforms, molecular docking

## Abstract

Digoxin and other cardiotonic steroids (CTS) exert their effect by inhibiting Na,K-ATPase (NKA) activity. CTS bind to the various NKA isoforms that are expressed in different cell types, which gives CTS their narrow therapeutic index. We have synthesised a series of digoxin derivatives (γ-Benzylidene digoxin derivatives) with substitutions in the lactone ring (including non-oxygen and ether groups), to obtain CTS with better NKA isoform specificity. Some of these derivatives show some NKA isoform selective effects, with BD-3, BD-8, and BD-13 increasing NKA α2 activity, BD-5 inhibiting NKA α1 and NKA α3, BD-10 reducing NKA α1, but stimulating NKA α2 and α3; and BD-14, BD-15, and BD-16 enhancing NKA α3 activity. A molecular-docking approach favoured NKA isoform specific interactions for the compounds that supported their observed activity. These results show that BD compounds are a new type of CTS with the capacity to target NKA activity in an isoform-specific manner.

## Graphical abstract

**Figure:**
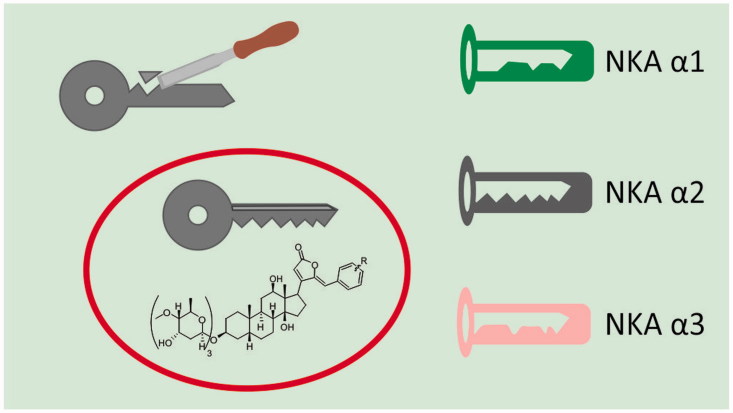

## Introduction

Na,K-ATPase (NKA) is an enzyme responsible for the transport of Na^+^ and K^+^ across the plasma membrane of most animal cells. NKA generates the electrochemical ion gradients that drive many different cell processes, including cell excitability, glucose uptake, and water and salt transport. NKA was discovered by the Danish researcher Jens Skou, who used the cardiotonic steroid (CTS) ouabain, derived from plants of the *Strophanthus* genus, to specifically define the NKA activity^1^. According to the most currently accepted mechanism of action of CTS, ouabain binds and locks NKA when in its E2-P conformation, a step in the NKA reaction cycle in which the enzyme has a high affinity for CTS^2^ ^,^ ^3^. In E2-P, NKA is phosphorylated, releases Na^+^ ions to the extracellular side of the cell plasma membrane, and is ready to bind and transfer K^+^ ions to the cytosol.

NKA is constituted by three different subunits: the α, β, and a smaller polypeptide, which depending on the tissue, consists of one of several members of the FXYD family of polypeptides^4^ ^,^ ^5^. The α subunit is responsible for the catalytic activity of NKA and contains the ATP, Na^+^, and K^+^ binding sites. The β subunit is a glycosylated polypeptide responsible for the folding and functional competence of the NKA α subunit. The FXYD peptide functions as a modulator of the catalytic properties of NKA. Four isoforms of the NKA α subunit and three NKA β isoforms have been identified in mammals (α1, α2, α3, α4, β1, β2, and β3). This molecular heterogeneity, in addition to various assemblies of the α and β subunits, provides cells with the versatility of adjusting Na^+^ and K^+^ gradients to the needs of each cell type^5^.

**Figure 1.**
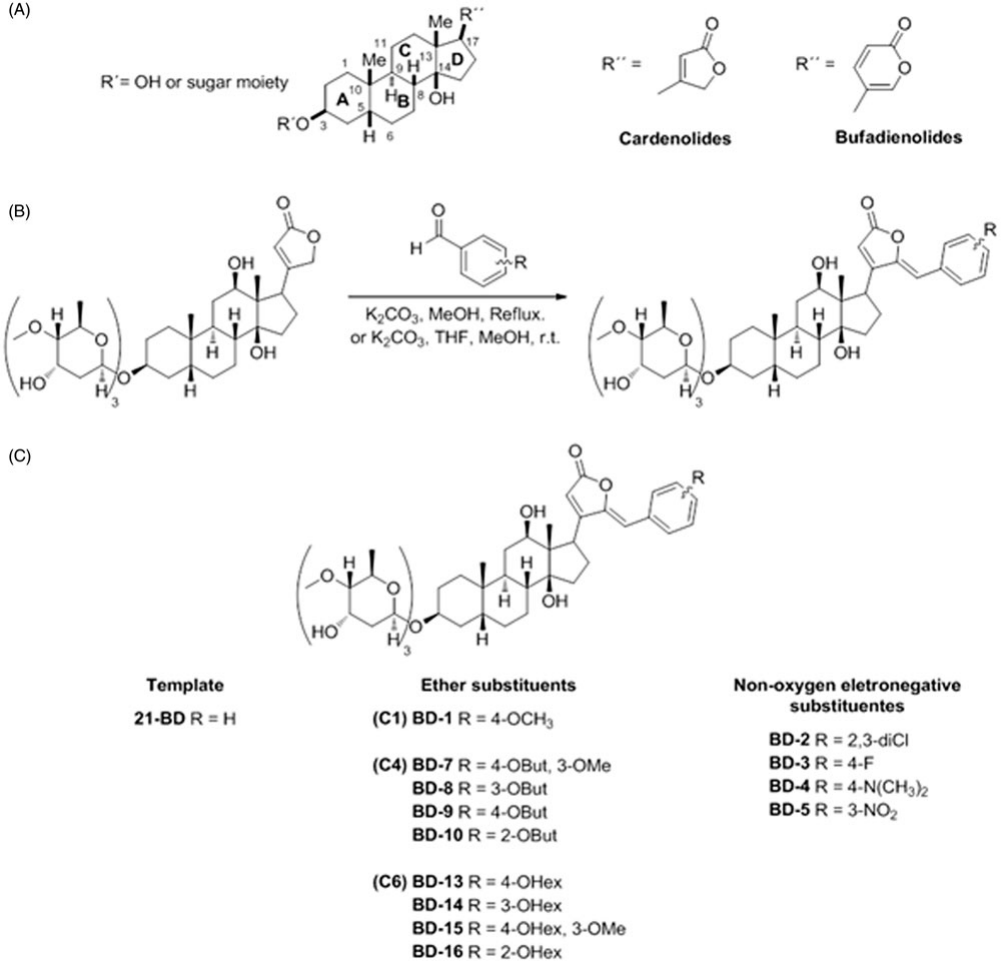
General scheme of the cardiotonic steroid structure (A), general procedure for the synthesis of digoxin derivatives (B), and group and subgroup split of derivatives according to their structure (C).

Due to their capacity of inhibiting NKA, CTS secondarily increase intracellular Ca^2+^ in myocardiocytes and cause higher cardiac contraction force and output. For this reason, CTS have been used in the treatment of congestive heart failure^4^. These compounds share a common structural characteristic, which is to have a conserved steroidal core with a five-membered lactone ring (in the case of cardenolides) or six-membered lactone ring (for the bufadienolides), attached to C17; and a sugar moiety, composed of one or more hydrocarbons (for the glycosides) or just a hydroxyl group (for the genins), linked to C3 (Figure 1(A))^4^ ^,^ ^6^.

Several studies have been conducted to determine the CTS binding site within the NKA. It has been shown that at least part of the ouabain binding site resides on the extracellular side of the NKA α isoform, comprising especially the extracellular loops between the TM1-TM2, TM5-TM6, and TM9-TM10 transmembrane domains^7^ ^,^ ^8^. However, other studies have reported that ouabain can also interact with the transmembrane domains TM5 and TM6, partially unwinding the TM4 helix^9^ ^,^ ^10^. The ouabain lactone ring has been shown to accommodate close to Val329 and Ala330 in the TM4 domain, which displaces Gly326, an amino acid residue that is crucial for K^+^ coordination^9^. The steroid core helps the molecule to interact with a hydrophobic pocket at TM4-TM6 domains guided mainly by Phe323, Phe790, and Phe793, whereas the sugar moiety appears to make hydrogen bonds with Glu319 and Arg887 on the TM7-TM8 loop and the TM4 domain, respectively^9^ ^,^ ^10^. Understanding how ouabain interacts with NKA is of high relevance, since it will help deciphering how other CTS differing on lactone ring, hydroxyl groups attached to the steroidal core, and sugar moieties interact with NKA. This information can then be used to develop compounds with higher binding capacity and potency.

In the 1990s, some researchers observed that ouabain exerted effects on NKA that were independent from inhibition of the enzyme and rather resulted from the activation of a cascade of intracellular events that caused cardiomyocyte hypertrophy^11–14^. This revealed a new function for the NKA and showed that it can function as a receptor and signal transducer that mediates the effects of ouabain in cells. This role of NKA has been the topic of intense research, with the goal of exploiting NKA signalling for its use in disease and cancer. Several laboratories have designed and generated, particularly via hemi-synthesis, novel CTS derivatives^15–17^. Our group is interested in developing new CTS derivatives which can exert effects that are more specific. We have generated a series of compounds by modifying the lactone ring of the CTS digoxin. One of these synthetic digoxin analogues, 21-benzylidene digoxin (21-BD), presented cytotoxic and antiproliferative effects when used on HeLa cancer cells, via inducing apoptosis in those cells, but inhibiting NKA activity just at high micromolar levels^18^ ^,^ ^19^. Subsequently, starting with the structure of digoxin, we synthesised a series of γ-benzylidene derivatives and showed that they have cytotoxic effects on HeLa and RKO cancer cell lines that are independent from the modulation of NKA activity^20^.

One of the disadvantages of the use of CTS is that they exhibit a very narrow safety margin and have toxic effects that are secondary to sustained NKA inhibition^21^. In addition, the ubiquitous presence of NKA in all tissues, along with a cell type specific pattern of expression of its various isoforms, all of which being able to respond to CTS, contribute to the widespread action of the CTS. In this manner, it is clear that there is a need for developing compounds that can more specifically target a particular NKA isoform type, which will help to prevent some of their secondary effects.

In this work, we show the synthesis of a series of new CTS compounds, derived from digoxin by introducing non-oxygen and ether substituents into the lactone ring. Analysis of these compounds on NKA activity and studies of their interactions with the NKA α isoforms using a molecular docking approach shows that some of these compounds exhibit isoform specific properties. This shows new data for the structural features underlying the interaction of CTS with NKA and provides new compounds with a better NKA isoform specificity.

## Material and methods

### General procedure for the synthesis of digoxin derivatives (21-BD), (BD-1), (BD-2), (BD-3), (BD-5), (BD-7), (BD-8), (BD-10), (BD-13), (BD-14), (BD-15), and (BD-16)

Aldehyde (1.8 mmol), digoxin (0.469 g, 0.6mmol), anhydrous K_2_CO_3_ (0.249 g, 1.8 mmol) were added to 60 ml of methanol in a round bottom flask. After stirring for 6 h at 70 °C, the solvent was evaporated in a rotary evaporator. The crude product was diluted with 20 ml of water and extracted with hot ethyl acetate (3 × 30 ml). The organic layer was washed with brine, dried over anhydrous Na_2_SO_4_ and concentrated under vacuum. The crude product was purified by silica column chromatography (CH_2_Cl_2_/MeOH 11:1). After purification, the pure product was diluted in THF, precipitated with hexane and concentrated under reduced pressure to give the benzylidene digoxin derivatives (Figure 1(B)).

### General procedure for the synthesis of digoxin derivatives (BD-4) and (BD-9)

Aldehyde (2 mmol), digoxin (0.781 g, 1.0 mmol), anhydrous K_2_CO_3_ (1.1 g, 7.96 mmol), were added to 4 ml of methanol and 2 ml THF in a round bottom flask. After stirring for 6 h at room temperature, the solvent was evaporated in a rotary evaporator. The crude product was diluted with 20 ml of water and extracted with hot ethyl acetate (3 × 30 ml). The organic layer was washed with brine, dried over anhydrous Na_2_SO_4_ and concentrated under vacuum. The crude product was purified by silica column chromatography (CH_2_Cl_2_/MeOH 11:1). After purification, the pure product was diluted in THF, precipitated with hexane and concentrated under reduced pressure to give the benzylidene digoxin derivatives as a yellow solid (Figure 1(B)).

### Insect cell culture and viral infections

Sf9 insect cells were grown in Grace’s medium with 3.3 g/l lactalbumin hydrolysate, 3.3 g/l yeastolate, and supplemented with 10% (v/v) fetal bovine serum, 100 units/ml penicillin, 100 µg/ml streptomycin, and 0.25 µg/ml fungizone. Cells were grown in suspension cultures and were transferred to 150 mm tissue culture plates before infection. Infections were performed as previously described, using baculoviruses carrying the desired NKA α isoform^22^.

### Membrane preparation

After 72 h of infection, cells were scraped from the culture plates, centrifuged at 1500×*g* for 10 min and suspended in 250 mM sucrose, 0.1 mM EGTA, and 25 mM imidazole HCl, pH 7.4, using a motor-driven Teflon Potter-Elvehjem homogenizer (Thomas Scientific, Swedesboro, NJ). The sample was then subjected to centrifugation at 4500×*g* for 10 min. The resulting supernatant was centrifuged at 70,000×*g* for 1 h. The final pellet was re-suspended in the homogenisation solution and used for NKA activity assays.

### NKA activity

The cell membrane preparations were incubated 20 min with digoxin derivatives. The reaction was carried on at 37 °C for 30 min in 120 mM NaCl, 20 mM KCl, 2 mM MgCl_2_, 3 mM ATPNa_2_, and 50 mM HEPES (pH 7.5), in the absence and presence of 1 mM ouabain (10 min of incubation). NKA activity was determined by measuring the released inorganic phosphate according to a colorimetric method described previously^23^, and specific activity was considered as the difference between the total and ouabain-treated samples ATPase activities (Table S2).

### Molecular docking

Initially, the 3D structures of ligands were generated by Marvin Beans 16.8.1.0 software (ChemAxon, Budapest, Hungary) in PDB format^24^, which protonated form and tautomeric conformers were carefully checked using pH 7.4^25^. Afterwards, all ligands were refined by semi-empirical Parametric Method 7 (PM7)^26^ implemented on MOPAC2012 using the keyword *ef* for minimum search^27^. Next, refined ligands in pdbqt format were signed to routable bonds, Gasteiger–Marsili net atomic charges^28^, and hydrogens from polar atoms were kept by MGLTools software^29^. The visual inspection of ligands geometry was performed by PyMOL application^30^.

In parallel, the amino acid primary sequence of NKA α1 (NP_036636.1), NKA α2 (NP_036637.1), and NKA α3 (NP_036638.1), deposited into National Center for Biotechnology Information (NCBI), was used to generate their 3D structure using Swiss-Model online software^31^ (Swiss Institute of Bioinformatics, Basel, Switzerland). All structures were built by homology modelling using the pig kidney NKA α1 isoform as a model (PDB ID code 4HYT)^32^. NKA α1 and α2 isoforms were built as precursors, containing the first five amino acids that are not found in mature NKA polypeptide. Then, these targets were prepared for molecular docking using MGLTools software^29^ (The Scripps Research Institute, La Jolla, CA). A grid box was built centred on CTS, covering up all binding sites, with −26.55, −20.197, and 71.573 for x, y, and z axis, respectively, and size of 20, 20, and 36 Å. The exhaustiveness was set to 20 to improve the search for best conformer into the binding site. Next, the ligands in pdbqt format were submitted to molecular docking process using the AutoDock Vina software (The Scripps Research Institute, La Jolla, CA)^33^. Ramachandran plots were used to validate the models and were generated by Procheck software (European Bioinformatics Institute, Hinxton, Cambridge, UK)^34^.

### Data analysis

The data were analysed using GraphPad Prism 5 (GraphPad Software, La Jolla, CA) and plotted as mean ± SEM. The mean values were compared using Two-way ANOVA, to compare the effects of increasing concentrations of the CTS and different NKA isoform assemblies in the NKA activity, followed by the Bonferroni *post hoc* test to indicate the source of the observed differences. Dose-response experiments were performed 3–6 times using different membrane preparation samples. NKA activity values at different concentrations of a particular compound and for each NKA isoform were normalised to the activity of the corresponding isoform in the absence of the compound. Statistical significance was set as *p* < .05.

## Results

### NKA activity

Sf9 cells were infected with baculoviruses driving the expression of the different NKA α isoforms (α1, α2, and α3) and the β1 isoform. Homogenates from the cells containing the different NKA isoforms were used to test the effects of the digoxin derivatives. As shown in Figure 1(C), to facilitate characterizing the effects and interaction of the compounds with different NKA isoforms, these derivatives were divided into three groups.

21-BD, considered the template compound, and the only molecule from this group, did not significantly affect the activity of NKA α2 and α3 isoforms (Figure S1(A)). Previous studies from our group have demonstrated that 21-BD inhibits the NKA activity derived from a mouse kidney membrane preparation, which only contains the NKA α1 isoform; and has no effect on rat brain NKA, which mainly expresses the NKA α2 and α3 isoforms^18^.

**Figure 2.**
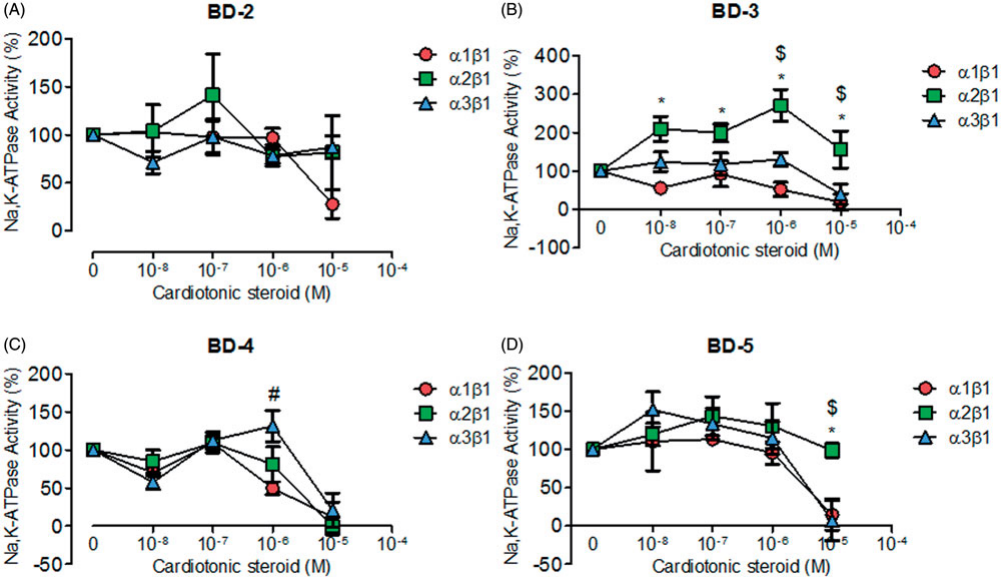
NKA activity (%) from Sf9 cell membrane preparations after BD-2 (A), BD-3 (B), BD-4 (C), and BD-5 (D) treatment for 20 min. *p < .05 differences between NKA α1β1 and α2β1 isoforms. $*p* < .05 differences between NKA α2β1 and α3β1 isoforms. #*p* < .05 differences between NKA α1β1 and α3β1 isoforms. Each point represents the mean ± SEM of at least three independent experiments performed in triplicate.

From a second group of compounds (BD-2, BD-3, BD-4, and BD-5), corresponding to chemical scaffolds with non-oxygen electronegative substituents in the aromatic ring level (Figure 2(A–D)), BD-2, similar to 21-BD, did not inhibit NKA α2 and α3 isoforms; however, it did reduce NKA α1 isoform activity at micromolar concentrations (Figure 2(A)). BD-3 exhibited selectivity for the NKA α2 isoform, increasing its activity over 100% at almost all concentrations tested. In contrast, BD-3 inhibited NKA α1 and α3 isoforms at high concentrations (Figure 2(A)). BD-4 shows no selectivity for the NKA isoforms, but it is able to inhibit all isoforms at micromolar concentrations (Figure 2(C)). Likewise, BD-5 exhibited no NKA isoform specific effects at relatively low concentrations, but interestingly it inhibited the NKA α1 and α3 isoforms and not NKA α2 at high concentrations (Figure 2(D)).

**Figure 3.**
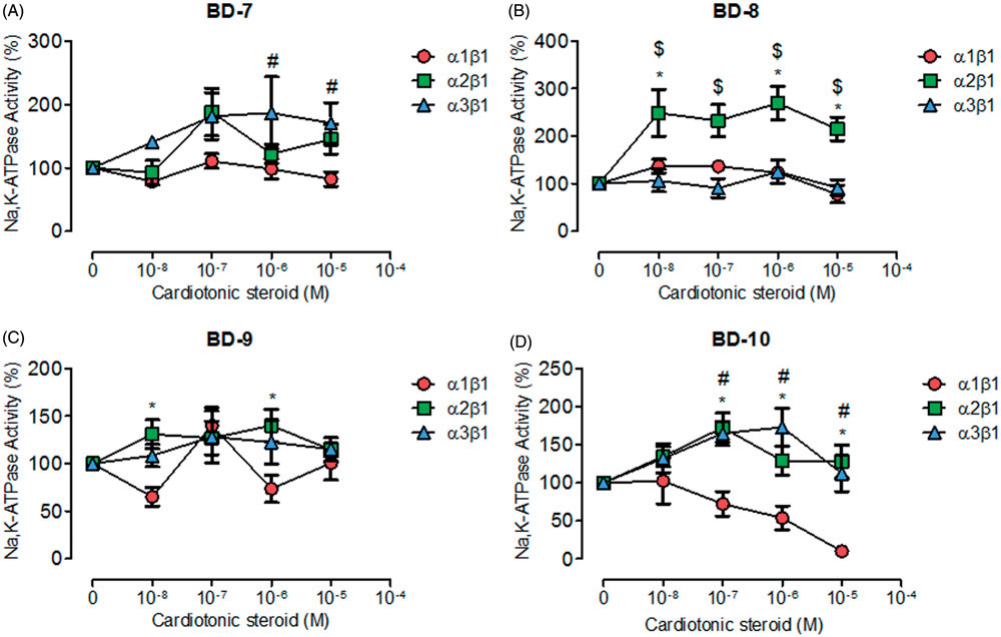
NKA activity (%) from Sf9 cell membrane preparations after BD-7 (A), BD-8 (B), BD-9 (C), and BD-10 (D) treatment for 20 min. *p < .05 differences between NKA α1β1 and α2β1 isoforms. $*p* < .05 differences between NKA α2β1 and α3β1 isoforms. #*p* < .05 differences between NKA α1β1 and α3β1 isoforms. Each point represents the mean ± SEM of at least three independent experiments performed in triplicate.

The third group of compounds that we synthesised, consisted in derivatives with ether radicals as functional groups, attached to the aromatic ring of the template 21-BD. These compounds could be further subdivided into three subgroups, according to their carbon chain length, containing one, four, and six-carbons (C1, C4, and C6, respectively). BD-1, the only C1 compound did not exhibit selectivity for any particular NKA α isoforms, nor had any effect on their activity (Figure S1(B)). Concerning the compounds of the C4 subgroup, BD-7 and BD-8 did not affect NKA α1 and α3 isoforms (Figure 3(A,B)). However, BD-8 significantly increased NKA α2 activity at all concentrations (Figure 3(B)). BD-9 showed no effect on NKA α1, α2 and α3 (Figure 3(C)). However, BD-10 exhibited a strong dose-response inhibitory curve for the NKA α1 isoform, but stimulated the NKA α2 and α3 isoforms at concentrations between 100 nM and 1 µM (Figure 3(D)).

**Figure 4.**
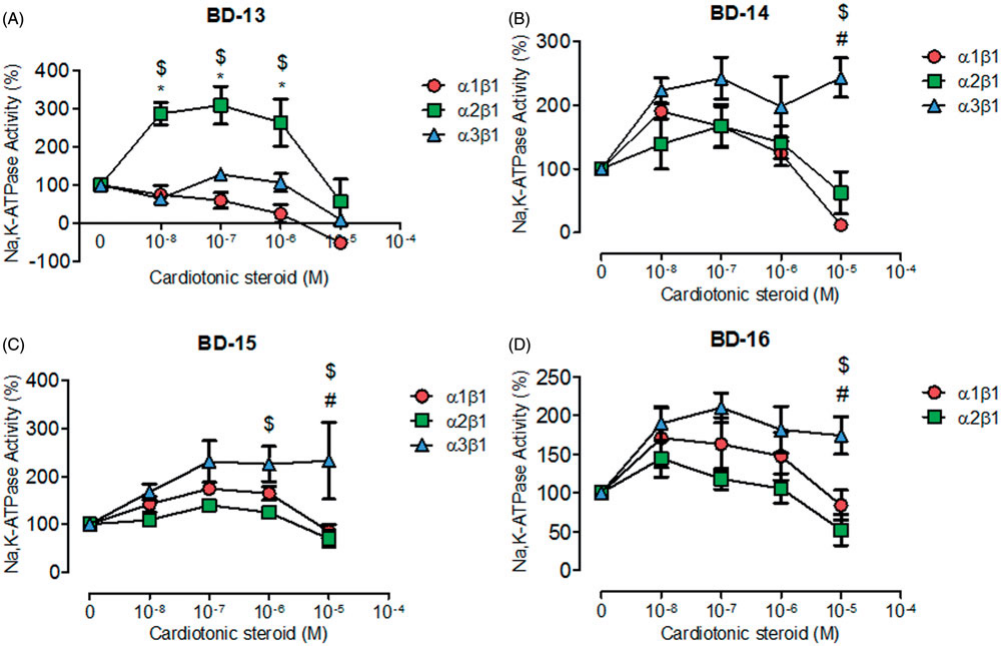
NKA activity (%) from Sf9 cell membrane preparations after BD-13 (A), BD-14 (B), BD-15 (C), and BD-16 (D) treatment for 20 min. *p < .05 differences between NKA α1β1 and α2β1 isoforms. $*p* < .05 differences between NKA α2β1 and α3β1 isoforms. #*p* < .05 differences between NKA α1β1 and α3β1 isoforms. Each point represents the mean ± SEM of at least three independent experiments performed in triplicate.

The compounds of subgroup C6, almost all showed a significant selectivity for NKA α3, stimulating its activity (Figure 4(A–D)). BD-13 was the only derivative that demonstrated a different effect, stimulating the NKA α2 isoform in the nanomolar range and inhibiting the NKA α1 and α3 isoforms in the micromolar range (Figure 4(A)). BD-14, BD-15, and BD-16 significantly stimulated the NKA α3 isoform at all concentrations, whereas they did not affect the NKA α2 isoform (Figure 4(B–D)). The effects of these last three compounds on the NKA α1 isoform showed some peculiarities, with BD-14 showing the dual effect observed for classical CTS (Figure 4(D)), whereas BD-15 also showed a stimulatory effect at nanomolar levels, but no inhibitory effect in the micromolar range (Figure 4(C)). BD-16 did not significantly affect either the NKA α1 or α2 isoforms (Figure 4(D)).

Altogether, these results suggest that changes in the chemical side groups attached to the lactone ring are able to modify the interaction of the resulting CTS with the NKA, in some cases in an isoform specific manner.

### Molecular docking

As a correlate to the experimental response of NKA isoforms to each of the synthetic CTS that we generated, we studied their interaction at the molecular level, with the goal of finding the basis for their different NKA effects. The rat NKA α1, α2, and α3 sequences show 96.87, 86.47, and 86.57% of identity, respectively, compared to the pig NKA α1 isoform^35–37^. Ouabain has been one of the most studied CTS, and therefore, we used it as a model in our molecular docking studies. Our synthetic derivatives, as well as ouabain, were docked on the rat NKA α subunit isoforms. First, the docked compounds were refined through a semi-empirical approach to correct geometric parameters, such as bond lengths. Initially, a re-docking with the refined ouabain ligand and the crystallographic ouabain on the pig NKA α1 isoform was performed^18^. This provided a root mean square deviation (RMSD) value of 2.24 Å for the best solution, supporting, along with the NKA α isoforms Ramachandran plots (Figures S2–S4), the accuracy of the methodology used. After this, selected compounds were subjected to molecular docking.

**Figure 5.**
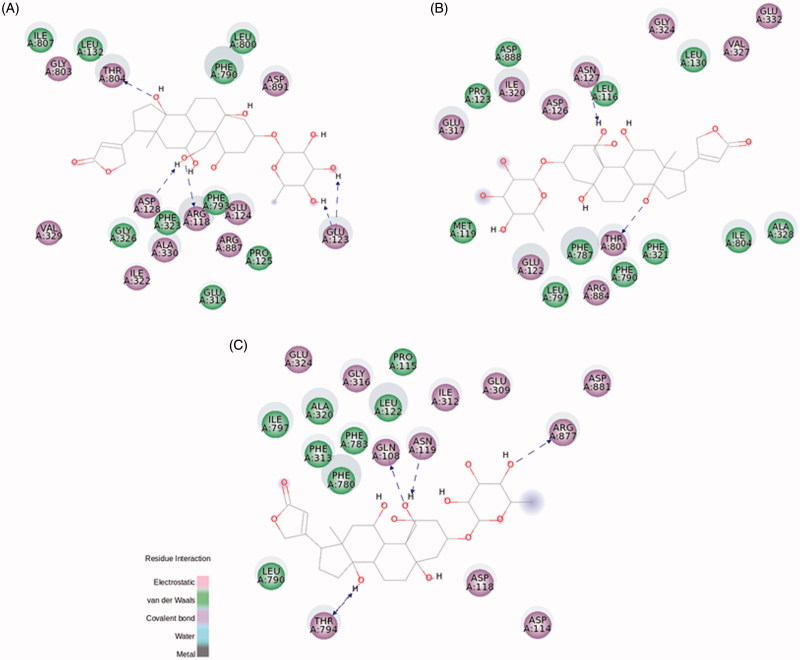
Ouabain molecular docking on rat NKA α1 (A), α2 (B), and α3 (C) isoforms. The green and magenta circles represent residues involved in van der Waals and polar interactions, respectively. The blue halo around the residue is proportional to the solvent accessible surface. Pi interactions are represented by an orange line and symbols indicating the specific interaction. The green dashed arrows are directed towards the electron donor and represent hydrogen bonds with amino acid main chains. The blue dashed arrows are directed towards the electron donor and represent hydrogen bonds with amino acid side-chains.

Ouabain docking showed a series of relevant interactions with the NKA α1 isoform (Figure 5(A)). The lactone ring interacts with Val329, responsible for the coordination of K^+^ ions, and with Gly803 interacting by electrostatic bonds. Close to the steroidal core and in agreement with previous results obtained with NKA from shark rectal gland, we found some interactions with phenylalanine residues (Phe323, Phe790, and Phe793), through van der Waals interactions^9^, as well as with Ala330 and Leu800. Hydrogen bonds are established between the C11α, C19α, and C14β of ouabain and the residues Arg118, Asp128, and Thr804, respectively. Arg118 has been identified as one of the key residues involved in the ouabain resistance of the rodent NKA α1 subunit^38^. Despite this has been related to an increase in the dissociation constant of the ouabain-rodent NKA α1 complex^39^ ^,^ ^40^, the binding energy of the docked ouabain with the three NKA α isoforms shows similar values (Table S1). At the rhamnose level, Glu123 forms hydrogen bonds with the distal hydroxyl groups.

Regarding NKA α2 and α3 isoforms, both of them are slightly smaller than NKA α1 (1020 and 1013 amino acids, respectively, compared to 1023 amino acids for α1 – Figure S5)^41^. Despite this, most of the interactions with ouabain are conserved, especially for the amino acids interacting with the steroidal core and the sugar moiety (Figure 5(B,C)). Some positional correspondences are observed, like the interaction with Arg118 for NKA α1, Leu116 for NKA α2, and Gln108 for NKA α3, but the residue exchange seems to be a key factor to the previously observed effect for the ouabain resistance^38^.

21-BD was used as the template compound for docking, due to the simplicity of its structure compared with the other derivatives. The presence of an aromatic ring attached to the lactone ring C21 helps the molecule to interact with a hydrophobic pocket formed by Ala330, Glu786, Phe790, Leu800, and Ile807 at NKA α1 isoform (Figure S6(A)). Thr804 makes hydrogen bond with a different hydroxyl group, attached to C12α, compared to ouabain. The presence of two extra distal sugar units showed new polar interactions with Asp892 and Arg893, as well as hydrogen bonds with Trp894. Indeed, all these different features observed molecularly, compared to ouabain, are evidenced by the absence of any modulatory effect of 21-BD, in nanomolar concentrations, on NKA activity^18^. Unlike ouabain, not many conserved interactions were found when the three NKA α isoforms were compared, with different residues making polar and nonpolar interactions with all the three CTS moieties (Figure S6(B,C)).

**Figure 6.**
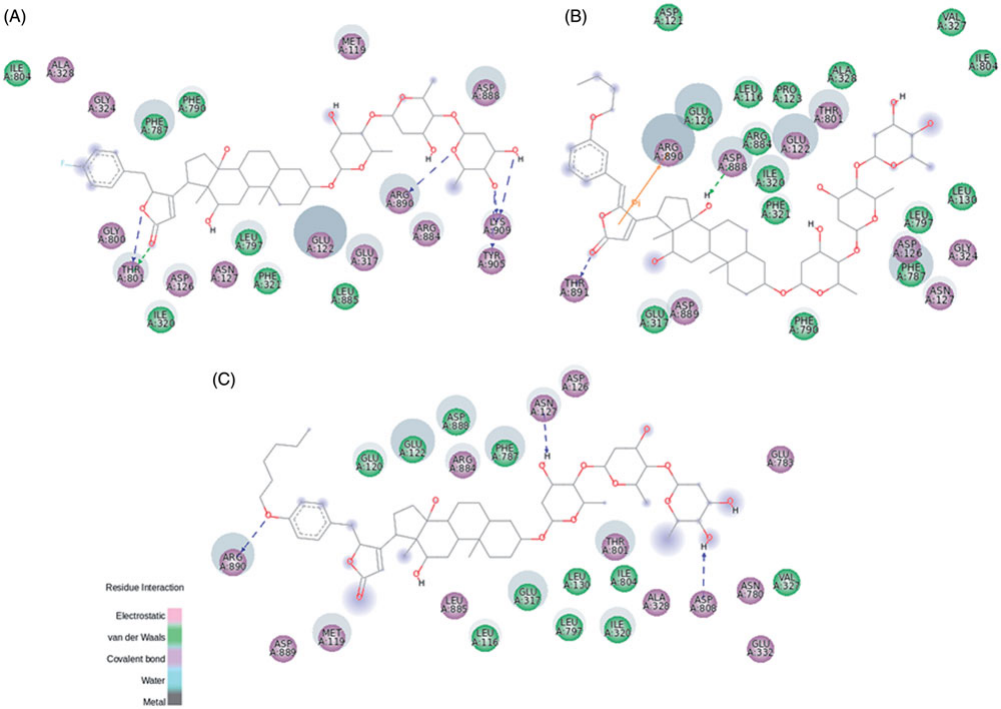
BD-3 (A), BD-8 (B), and BD-13 (C) molecular docking on rat NKA α2 isoform. The green and magenta circles represent residues involved in van der Waals and polar interactions, respectively. The blue halo around the residue is proportional to the solvent accessible surface. Pi interactions are represented by an orange line and symbols indicating the specific interaction. The green dashed arrows are directed towards the electron donor and represent hydrogen bonds with amino acid main chains. The blue dashed arrows are directed towards the electron donor and represent hydrogen bonds with amino acid side-chains.

In BD-3, the representative compound of the non-oxygen electronegative substituents group, the fluorine addition at *p*-position of the aromatic ring rearranges the hydrophobic pocket that accommodates this nonpolar group. Also, some electrostatic contacts are found at this level (Figures 6(A) and S7). The electronegative characteristic of fluorine, evidenced by a bulky electronic cloud, keeps the linearity of BD-3 structure into the binding site, compared to a twisted shape of 21-BD docked on NKA α1 and α3 isoforms (Figure S7). BD-3 drastically increased the NKA α2 activity (Figure 2(B)). The interaction between Thr804 in NKA α1 isoform and C12α is displaced to the lactone ring, compared to the corresponding residue Thr801 of NKA α2 isoform. However, what is really different in this compound compared to 21-BD, and could explain BD-3 ability to increase NKA α2 activity, is the absence of a coordinative sigma–π interaction between Phe787 and the aromatic ring (Figures 6(A) and S6(B)). Moreover, some interactions have changed their characteristics, even though the corresponding residues keep the same, as Ala328, Gly324, and Gly800, all of them making polar interactions with BD-3, unlike to nonpolar interactions with 21-BD.

**Figure 7.**
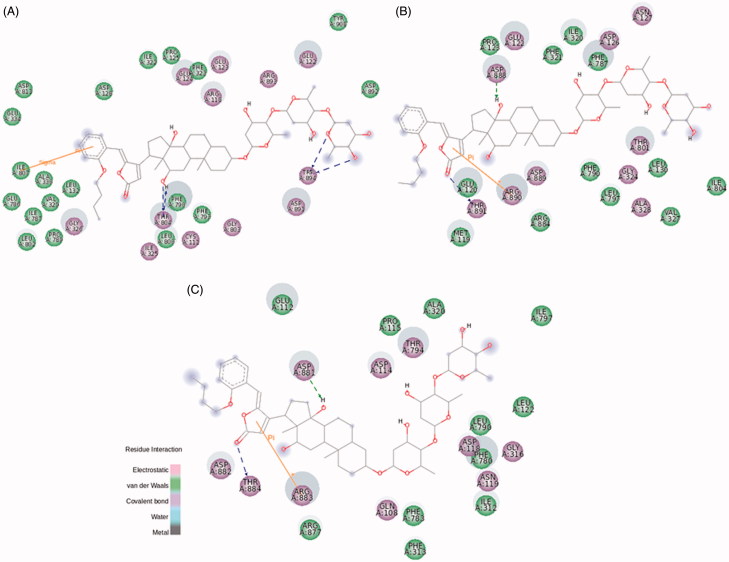
BD-10 molecular docking on rat NKA α1 (A), α2 (B), and α3 (C) isoforms. The green and magenta circles represent residues involved in van der Waals and polar interactions, respectively. The blue halo around the residue is proportional to the solvent accessible surface. Pi interactions are represented by an orange line and symbols indicating the specific interaction. The green dashed arrows are directed towards the electron donor and represent hydrogen bonds with amino acid main chains. The blue dashed arrows are directed towards the electron donor and represent hydrogen bonds with amino acid side-chains.

The ether radical derivatives, BD-8 and BD-10 present a butoxy group attached to the aromatic ring at *m*- and *o*-position, respectively. Both compounds conserved the interactions between Thr804 and C12α hydroxyl group, and hydrogen bonds between Trp894 and the distal digitoxose unit, when compared to the template 21-BD (Figures 6(B) and S8 for BD-8; Figure 7 for BD-10). Interestingly, BD-10 shows the classical inhibitory profile of CTS on NKA α1 activity, whereas this effect is not observed by BD-8. The presence of a butoxy group at *o*-position clearly drives a distortion at the C17-C20 bond to better fit the butoxy group at a hydrophobic portion of NKA α1 isoform (Figure 7(A)). Despite both compounds show the same binding energy for the interaction with NKA α1 (Table S1), many hydrophobic interactions are showed for BD-10, especially stabilizing *o*-butoxy-benzylidene group. Leu132, Val329, Ala330, Glu334, Glu786, Ile787, and additionally, Pro789 and Leu802 are found surrounding this substituent group for BD-10, whereas the possible corresponding residues are spread throughout the BD-8 substituent (Figure S8(A)). These stable hydrophobic contacts between BD-10 and NKA α1, together with a sigma–π interaction between Ile807 and the aromatic ring could explain the inhibitory capacity for this compound.

The addition of a butoxy group to 21-BD drastically changes the interaction with the NKA α2 isoform. Stable polar interactions made by Asp888 and Arg890 at distal digitoxose unit for 21-BD are wondrously found interacting by a cation–π and hydrogen bond at lactone ring and steroidal core level, respectively (Figures 6(B) and 7(B)). In fact, 21-BD presents a more favourable binding energy compared to BD-8 and BD-10 (Table S1), and this possibly disturbs the binding site for these compounds, favouring an increased flexibility of the enzyme and producing the increased NKA α2 activity that we observed. Looking into interactions of the compounds to NKA α3 isoform, BD-10 seems to displace residues from sugar moiety to lactone ring level, which could explain the slight increase on NKA α3 activity (Figure 7(C)). BD-8 did not induce this conformational change, and indeed, presents stable interactions at lactone and aromatic ring like 21-BD (Figure S8(B)).

The three derivatives from the C6 subgroup (BD-13, BD-14, and BD-15) present a hexoxy group attached to the aromatic ring: at *p*-position for BD-13 and BD-15, and at *m*-position for BD-14. The difference between BD-13 and BD-15 is an additional methoxy group at *m*-position for BD-15, similar to BD-7 from C4 subgroup. BD-13 was able to inhibit NKA activity reconstituted with NKA α1 and α3 isoforms just at micromolar levels, whereas a huge selectivity to the NKA α2 isoform was observed, increasing NKA activity at sub-micromolar concentrations (Figure 4(A)). The addition of a six-membered carbonic chain to the aromatic ring helps the molecule to interact with a hydrophobic pocket as stated before, and indeed, the threonine residue (Thr804 for NKA α1 isoform and Thr794 for NKA α3 isoform) forming a hydrogen bond with C12α of 21-BD is otherwise found interacting with the lactone ring and aromatic ring of NKA α1 and α3, respectively, for BD-13 (Figure S9). These changes and other contacts like a cation-π interaction between Arg118 and the lactone ring on NKA α1 isoform, and a sigma–π interaction between Phe780 and the aromatic ring on NKA α3 isoform, could partially stabilise the NKA-CTS complex and explains the ability of BD-13 to inhibit these two isoforms at micromolar range. In contrast, BD-13 did not inhibit, but increased NKA α2 activity at submicromolar levels. Accordingly, the binding energy for BD-13/NKA α2 complex is slightly high (Table S1). In searching for interactions that could explain this activation effect, a completely different interaction pattern for BD-13 and NKA α2 was identified (Figure 6(C)). Arg890, that was involved in hydrogen bond with the distal sugar unit of 21-BD, is placed at the opposite side of BD-13, making a hydrogen bond with the ether radical oxygen. Any kind of π interaction appears to aid in the stabilisation of the complex. The classical phenylalanine residues covering up the steroidal core (Phe321, Phe787, and Phe790 for NKA α2 isoform) were not observed for BD-13 and some residues, as Glu332, Asn780, Glu783, and Asp808, are involved with polar interactions at sugar moiety level and were not found for any other derivatives. Pedersen et al. have demonstrated that Asn776 from pig kidney NKA α1 is very important in Na^+^ coordination, and indeed, positional changes in its carboxamide group drastically decreases Na^+^ binding^42^. It is possible that the interaction with the corresponding residue Asn780 in rat NKA α2 could favour Na^+^ binding and explain the increase in activity that BD-13 causes on NKA α2. Mutations at Asp804 were described to almost completely abolish pig kidney NKA α1 activity by affecting the cation binding site^43^. However, Koenderink et al. have reported that some mutations on rat NKA α1 isoform may increase basal NKA activity, and postulated that the presence of Na^+^ at the cation binding pocket after the dephosphorylation step, quickly shifting the enzyme to E1 conformation is involved in this effect^44^. We have found a hydrogen bond between the corresponding residue (Asp808) in rat NKA α2 and the distal digitoxose unit of BD-13, suggesting that the lack of K^+^ coordination could favour a quick conformational shift of the enzyme, which can increase NKA activity. Tepperman et al. have demonstrated that Glu327 residue (corresponding to Glu332 in this study) is deeply involved with the first K^+^ occlusion^45^, and we have observed this residue involved with polar interactions at sugar moiety level.

Concerning BD-14 and BD-15, both of them have not presented any effect on NKA α2 activity (Figure 4(B,C)). On NKA α1 isoform, BD-14 has demonstrated a well-defined steroidal core attachment on Phe790, Phe793, and Leu800, hydrogen bonds formed between Thr804 and C12α and, these contacts already seen for BD-10 may explain the ability of BD-14 to inhibit this isoform at micromolar range (Figure S10(A)). Instead of that, the large carbon chain keeps barely accommodated, and indeed, it is not possible to observe a well-defined hydrophobic pocket for the BD-13/NKA α1 isoform complex. This statement, and a smaller binding energy compared to BD-13 (Table S1) could explain why BD-14 is able to increase NKA α1 activity at nanomolar concentrations. Regarding BD-15, the hydrogen bond between Thr804 and C12α was not observed, and phenylalanine residues covering up the steroidal core moves together with Thr804, guided by interactions with the lactone ring and, therefore, this molecule is not able to inhibit NKA α1 activity such as BD-14 does (Figure S11(A)).

**Figure 8.**
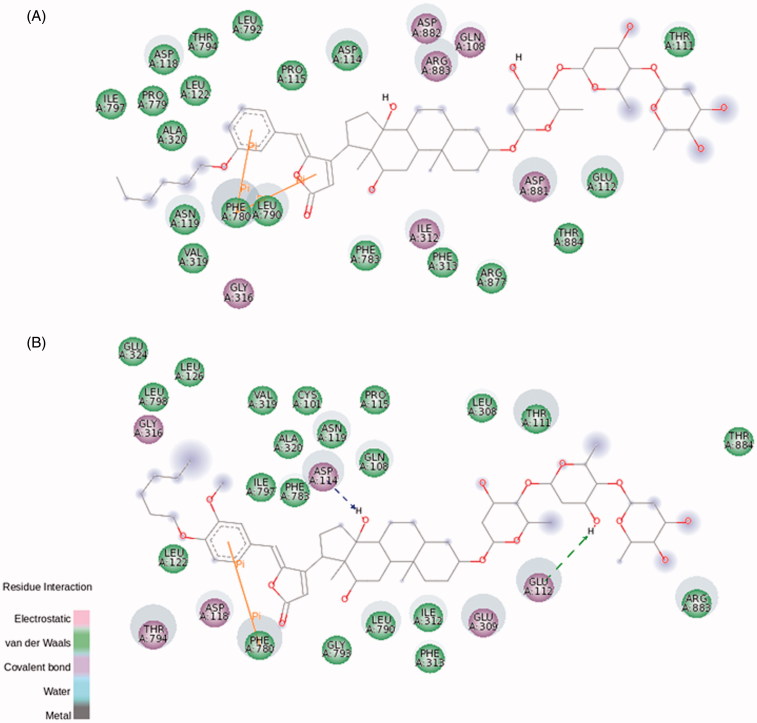
BD-14 (A) and BD-15 (B) molecular docking on rat NKA α3 isoform. The green and magenta circles represent residues involved in van der Waals and polar interactions, respectively. The blue halo around the residue is proportional to the solvent accessible surface. Pi interactions are represented by an orange line and symbols indicating the specific interaction. The green dashed arrows are directed towards the electron donor and represent hydrogen bonds with amino acid main chains. The blue dashed arrows are directed towards the electron donor and represent hydrogen bonds with amino acid side-chains.

Finally, BD-14 and BD-15 clearly demonstrated an important selectivity towards the NKA α3 isoform, increasing NKA activity at all concentrations (Figure 4(B,C)). The aromatic ring from both compounds and Phe780 are involved in π interactions. Residues involved with steroidal core interactions are totally displaced towards the lactone and aromatic ring, and almost all polar interactions at sugar moiety level are abolished (Figure 8). Weigand et al. have studied the NKA structural determinants for NKA α isoform selectivity of well-known digitalis-like compounds and, specifically, they have demonstrated a reduction in digoxin affinity for human NKA α2 isoform under pointed mutations at Met119, specifically, Met119Thr, Met119Asp, Met119Lys, Met119Asn, and Met119Ser^46^. Interestingly, the Met119Thr mutation was designed changing methionine residue for the corresponding threonine at NKA α1 isoform that also matches with threonine residue present at the same position of rat NKA α3 isoform. Therefore, a totally different pattern of CTS docking, together with threonine interaction during the pre-binding state^46^, could relieve CTS-NKA interaction and support an increased NKA α3 activity.

## Discussion

Since the studies of the British physician William Withering on the beneficial and toxic effects of the foxglove (*Digitalis purpurea*), CTS have been used for the treatment of congestive heart failure. However, because CTS bind to the NKA isoforms that are expressed throughout the body, they show a narrow therapeutic index and need to be carefully used in medicine. This stresses the need for compounds that can specifically target a single type of NKA isoform and encourage the synthesis of new CTS derivatives, with the goal of obtaining compounds with less global effects. Elbaz et al. have demonstrated the ability of a synthetic monosaccharide digitoxin analogue, D6-MA, to inhibit NKA activity and decrease cell viability in lung cancer cells^15^. Mijatovic et al. have synthesised a modified version of 2-oxovoruscharin, UNBS1450, with potent anti-cancer activity and also a great inhibitory effect on NKA activity^17^. Jensen et al. have synthesised mono and bivalent CTS using sulphur linked ethylene glycol moieties of varying length and they have shown their ability to inhibit NKA activity and their cytotoxic effect on the MCF-7 cancer cell line^16^. In previous work, we reported the synthesis of 21-BD by the addition of an aromatic ring to C17 of digoxin through a vinylogous aldol reaction. 21-BD was able to reduce the viability of cancer cells inhibiting the NKA activity just at high concentrations^18^. This data demonstrated a possible selective antitumor effect overcoming the toxic effects produced by NKA inhibition. Indeed, several authors have reported the ability of CTS to bind to NKA at nanomolar concentrations, without affecting its activity, but triggering signalling pathways inside the cell^12^ ^,^ ^14^ ^,^ ^47^ ^,^ ^48^. In this work, in an attempt to identify compounds with a selective NKA isoform modulation, we have performed the synthesis of novel digoxin analogues, adding different functional groups to the aromatic ring of 21-BD. We have evaluated their ability to modulate the NKA activity and characterised their interactions with NKA, using a molecular docking approach.

The selectivity of CTS for human NKA α isoforms has been studied and have been assigned to differences in sugar moiety^49^ ^,^ ^50^. Recently, it has been shown that some digoxin derivatives, with modifications at the third digitoxose, have a selectivity for NKA α2β1 and α2β3, compared to NKA α1β1, with up to 7.5 and 33.0 fold, respectively^51^. Natural CTS have also been shown to display a NKA α isoform selectivity in rat aorta and cultured cells in the absence and presence of K^+50^,^^52^^,^^53^^. Here, we show that changes in the lactone ring also alter the 21-BD selectivity for NKA α isoforms, either stimulating or inhibiting NKA in an isoform specific manner. These findings are important to help understand the specific interactions of CTS with NKA α isoforms and the consequences of their use in cell physiology.

Since 1985, several studies have identified the main amino acids in NKA that interact with CTS and the long-range structural changes that CTS drive in the protein^7^ ^,^ ^54^. These results originally come from random mutagenesis and site-directed mutation analyses^38^ ^,^ ^55^ ^,^ ^56^. More recently, NKA crystals were obtained^10^ under various conditions, such as the low-affinity ouabain form E2.2 K^+^.MgF_4_ ^2^−.ouabain^9^ and the high-affinity ouabain bound form E2P.nH^+^.ouabain state^57^. It has been shown that the ouabain lactone ring becomes in close proximity with Val329 and Ala330 at the TM4 helix, displacing Gly326 and partially unwinding this helix^9^. The steroidal core makes nonpolar interactions with three conserved phenylalanine residues (Phe323, Phe790, and Phe793) and the C14β forms a hydrogen bond with Thr804. The rhamnose group can make hydrogen bonds with Glu319 and Arg887^9^ ^,^ ^10^. A recent work performed by the Fedosova group has shown that digoxin binding to crystallised NKA from pig kidney involves the formation of a hydrogen bond between Thr804 and the C14β hydroxyl group of the CTS^58^. Our work shows that the simple addition of an aromatic ring in the digoxin lactone moiety shifts 21-BD in the binding site, stabilizing this hydrogen bond with C12 hydroxyl group on the α surface of the steroidal core (Figure S6(A)). Sugar moiety interactions present a huge difference between digoxin^58^ and 21-BD. Actually, some residues are changed in ouabain-resistant rat NKA α1 isoform and, in fact, some interactions at sugar moiety level seems to be more conserved compared with ouabain-sensitive rat NKA α2 isoform, such as the corresponding residues Glu122, Glu317, and Arg884 (Figure S6(B)).

Through the use of different mutagenesis approaches, the role of several amino acids in the coordination of Na^+^ and K^+^ ions has been demonstrated. Some of these mutations (including substitutions in Asp804) have been even shown to increase the NKA activity^42–45^. One of the likely mechanisms for this enhancement of the NKA activity was explained by Koenderink et al., who suggested that, in the mutants, Na^+^ is already present in the enzyme right after the dephosphorylation step, allowing a quick shift in conformation for the enzyme to start a new reaction cycle^44^. A similar mechanism was proposed by Tepperman et al., who have shown alterations in the kinetic parameters of a mutated NKA α2 isoform derived from rats^45^. We have found that some of our compounds interact with amino acids, which have been shown to be involved in NKA ion coordination. Therefore, it is possible that those compounds favour a conformational state similar to that induced by the mutations described in the references mentioned above^42–45^. In this manner, different from the effect of classical CTS, which cause steric blockage of the access of ions from the extracellular aqueous face to the ion binding sites, our NKA activating compounds may allow a rapid access of ions to their enzyme binding sites. It is clear that to assess these possibilities, additional experiments will be needed.

In conclusion, we have made a series of new digoxin derivatives by making substitutions in the lactone ring that exert NKA α isoforms selective effects, either stimulating or inhibiting NKA activity. These findings further advance our understanding of the molecular mechanisms underlying CTS interaction with NKA and open the possibility for the specific targeting of NKA isoforms with CTS. This will aid in further drug design, with the idea of reducing the side and toxic effects that CTS present.

## Supplementary Material

IENZ_1380637_Supplementary_Material.pdf
