# Supplementary material for: Selectivity analyses of γ-benzylidene digoxin derivatives to different Na,K-ATPase α isoforms: a molecular docking approach
Source: J Enzyme Inhib Med Chem. 2017 Nov 8;33(1):85–97. doi: 10.1080/14756366.2017.1380637 (PMC6009882; doi:10.1080/14756366.2017.1380637)
Supplement: IENZ_1380637_Supplementary_Material.pdf [file IENZ_A_1380637_SM8502.pdf]

Selectivity Analyses of γ-Benzylidene Digoxin Derivatives to Different Na,K-ATPase α Isoforms: A Molecular Docking Approach

Marco T. C. Pessôa[1], Silmara L. G. Alves[2], Alex G. Taranto[3], José A. F. P. Villar[2], Gustavo Blanco[4]*, and Leandro A. Barbosa[1]*

[1]Laboratório de Bioquímica Celular; Universidade Federal de São João del Rei, Campus Centro-Oeste Dona Lindú, Av Sebastião Gonçalves Coelho 400, 35501-296, Divinópolis, Brazil

[2]Laboratório de Síntese Orgânica e Nanoestruturas; Universidade Federal de São João del Rei, Campus Centro-Oeste Dona Lindú, Av Sebastião Gonçalves Coelho 400, 35501-296, Divinópolis, Brazil

[3]Laboratório de Modelagem Molecular; Universidade Federal de São João del Rei, Campus Centro-Oeste Dona Lindú, Av Sebastião Gonçalves Coelho 400, 35501-296, Divinópolis, Brazil

[4]Department of Molecular and Integrative Physiology, University of Kansas, Medical Center, 3901 Rainbow Blv. Kansas City, Kansas 66160

SPECTROSCOPY DATA

21-Benzylidene digoxin (**21-BD**)

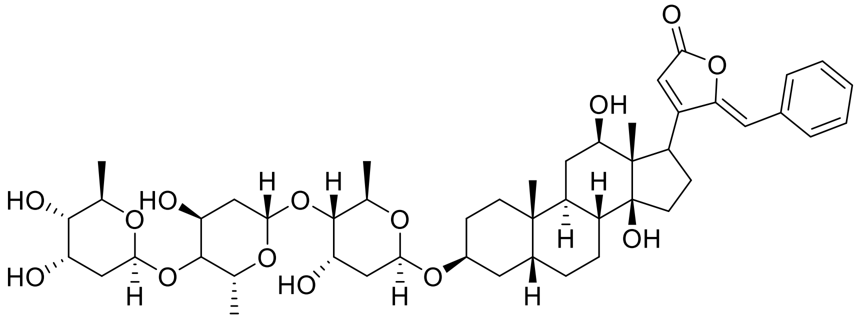

White solid, 0.33 g (0.37 mmol), yield 62%; m.p. 166-170 °C; IR (KBr) υ_{max}/cm^{-1} 3462, 2932, 2887, 1741, 1695, 1603, 1450, 1379, 1317, 1273, 1165, 1068, 1013, 870; ^{1}H NMR (400 MHz, DMSO) δ (ppm) 0.66 (s, 3H), 0.85 (s, 3H), 1.09-1.14 (m, 9H), 1.27-1.88 (m, 25H), 3.00 (ddd, *J* = 2.94 Hz, *J* = 6.87 Hz, *J* = 9.54 Hz, 2H), 3.11-3.15 (m, 3H), 3.61-3.75 (m, 5H), 3.84-3.93 (m, 3H), 4.20 (d, *J* = 2.16 Hz, 1H), 4.26 (d, *J* = 2.16 Hz, 1H), 4.59-4.63 (m, 3H), 4.79-4.83 (m, 3H), 5.00 (d, *J* = 5.47 Hz, 1H), 6.20 (s, 1H), 6.76 (s, 1H), 7.36-7.38 (m, 1H), 7.45 (t, *J* = 7.56 Hz, 2H), 7.67-7.70 (m, 2H); ^{13}C NMR (100 MHz, DMSO) δ (ppm) 169.18, 166.47, 149.78, 133.07, 130.03, 128.82, 128.70, 114.76, 110.05, 98.92, 98.83, 95.21, 84.93, 81.78, 81.52, 73.57, 72.55, 71.99, 68.95, 67.51, 67.39, 66.91, 66.15, 66.01, 59.65, 55.68, 41.84, 38.30, 38.19, 37.78, 36.23, 34.60, 32.27, 31.61, 30.07, 29.86, 29.51, 28.90, 26.38, 25.93, 23.56, 21.28, 18.24, 17.92, 13.99, 9.31; HRMS (m/z): [M+Na]$^{+}$ 891.4505 (calculated 891.4501) calculated for $C_{48}H_{68}NaO_{14}^{+}$.

21-*p*-Methoxylbenzylidene digoxin (**BD-1**)

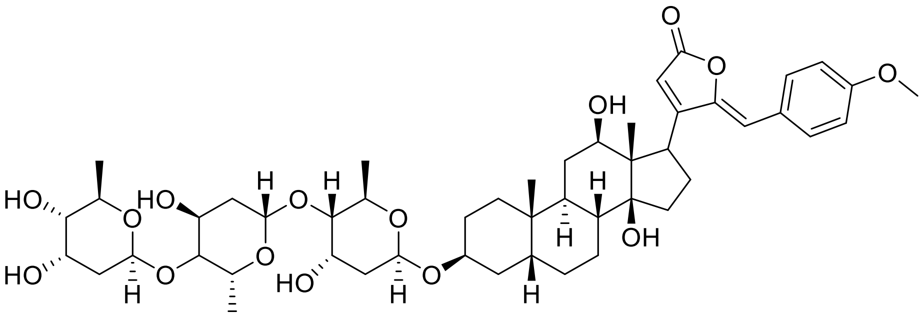

White solid, 0.48 g (0.53 mmol), yield 88%; m.p. 124-132 °C; IR (KBr) υ_{max}/cm^{-1} 3447, 2934, 1736, 1695, 1605, 1448, 1383, 1304, 1252, 1165, 1067, 1013, 870; ^{1}H NMR (400 MHz, DMSO) δ (ppm) 0.64 (s, 3H), 0.85 (s, 3H), 1.08-1.13 (m, 9H), 1.28-1.89 (m, 25H), 3.00 (ddd, *J* = 2.85 Hz, *J* = 6.96 Hz, *J* = 9.51 Hz, 2H), 3.10-3.15 (m, 3H), 3.60-3.73 (m, 5H), 3.73 (s, 1H), 3.80 (s, 2H), 3.83-3.93 (m, 3H), 4.21-4.23 (m, 1H), 4.27-4.29 (m, 1H), 4.62-4.65 (m, 3H), 4.79-4.83 (m, 3H), 4.99 (d, *J* = 5.45 Hz, 1H), 6.13 (s, 1H), 6.70 (s, 1H), 7.03 (d, *J* = 8.98 Hz, 2H), 7.64 (d, *J* = 8.92 Hz, 2H); ^{13}C NMR (100 MHz, DMSO) δ (ppm) 169.41, 166.41, 159.68, 148.31, 131.81, 127.32, 125.74, 115.75, 114.47, 113.71, 110.15, 98.98, 95.23, 84.91, 84.26, 81.82, 81.57, 73.63, 72.91, 72.58, 72.02, 68.98, 67.54, 66.93, 66.19, 59.70, 55.64, 55.23, 45.10, 41.84, 38.22, 37.81, 36.25, 34.62, 32.31, 31.63, 29.89, 29.52, 28.99, 26.72, 26.41, 25.94, 23.59, 21.32, 20.70, 18.28, 17.95, 14.02, 9.34; HRMS (m/z): [M+Na]$^{+}$ 921.4602 (calculated 921.4607) calculated for $C_{49}H_{70}NaO_{15}^{+}$.

21-2,3-Dichlorobenzylidene digoxin (**BD-2**)

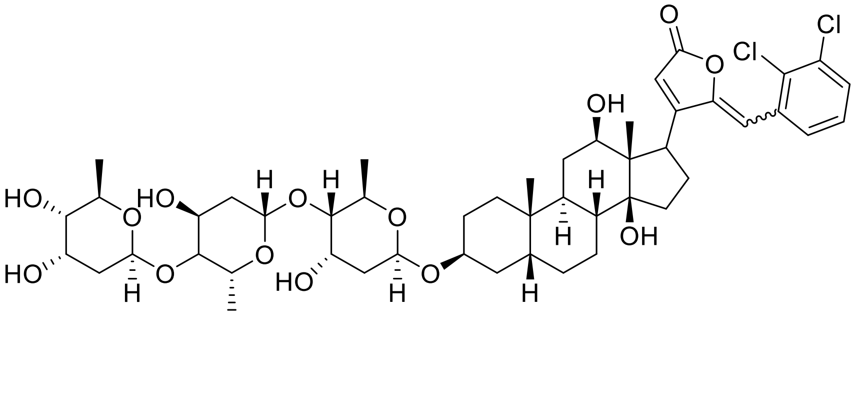

White solid, 0.56 g (0.60 mmol), yield 99%; m.p. 164-170 °C; IR (KBr) υ_{max}/cm^{-1} 3462, 2934, 2880, 1744, 1697, 1601, 1454, 1379, 1317, 1275, 1165, 1067, 1013, 868, 725; ^{1}H NMR (400 MHz, $CDCl_3$) δ (ppm) 0.77 (s, 3H), 0.92 (d, *J* = 3.36 Hz, 3H), 1.20-1.30 (m, 9H), 1.42-2.18 (m, 25H), 3.00 (s, 1H), 3.07 (s, 1H), 3.19 (dd, *J* = 2.87 Hz, *J* = 9.42 Hz, 1H), 3.24 (dd, *J* = 2.96 Hz, *J* = 9.32 Hz, 1H), 3.26-3.29 (m, 1H), 3.38 (t, *J* = 8.54 Hz, 1H), 3.45 (t, *J* = 8.32 Hz, 1H), 3.52-3.58 (m, 1H), 3.72-3.85 (m, 4H), 4.02 (s, 1H), 4.08-4.11 (m, 1H), 4.22-4.26 (m, 2H), 4.42-4.47 (m, 1H), 4.83-4.92 (m, 4H), 6.61-6.99 (m, 1H), 7.18 (dt, *J* = 4.28 Hz, *J* = 7.88 Hz, *J* = 7.94 Hz, 1H), 7.25 (s, 1H), 7.30 (s, 1H), 7.44 (dd, *J* = 1.32 Hz, *J* = 8.06 Hz, 1H); ^{13}C NMR (100 MHz, $CDCl_3$) δ (ppm) 202.52, 202.22, 198.20, 198.08, 145.96, 145.09, 133.87, 133.15, 133.04, 130.48, 127.60, 98.23, 95.42, 86.55, 82.54, 82.17, 76.33, 72.66; 72.53, 69.49, 68.23, 68.08, 66.45, 66.34, 56.74, 56.55, 42.73, 42.54, 41.65, 41.51, 37.81, 37.11, 36.70, 36.23, 35.01, 33.28, 32.28, 30.29, 30.13, 29.82, 29.64, 29.25, 28.59, 27.47, 26.58, 26.47, 23.51, 21.79, 18.14, 9.37, 9.25; HRMS (m/z): $[M+H]^+$ 937.3905 (calculated 937.3902) calculated for $C_{48}H_{67}Cl_2O_{14}^+$.

21-*p*-fluorbenzylidene digoxin (**BD-3**)

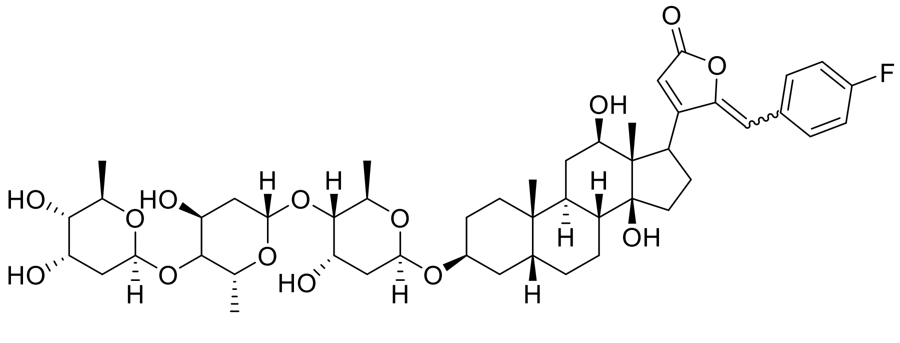

White solid, 0.36 g (0.40 mmol), yield 67%; m.p. 126-132 °C; IR (KBr) υ_{max}/cm^{-1} 3443, 2934, 2883, 1742, 1697, 1601, 1450, 1369, 1317, 1273, 1163, 1067, 1014, 870; ^{1}H NMR (400 MHz, $CDCl_3$) δ (ppm) 0.70 (s, 3H), 0.74 (s, 3H), 0.87-0.93 (m, 17H), 1.20-1.30 (m, 87H),

1.41-2.18 (m, 138H), 3.20 (dd, *J* = 2.77 Hz, *J* = 9.43 Hz, 5H), 3.24 (dd, *J* = 2.97 Hz, *J* = 9.22 Hz, 5H), 3.28 (dd, *J* = 3.11 Hz, *J* = 9.52 Hz, 5H), 3.43-3.37 (m, 3H), 3.53-3.58 (m, 3H), 3.65-3.86 (m, 18H), 3.96 (s, 1H), 4.08-4.11 (m, 4H), 4.22-4.26 (m, 8H), 4.83-4.92 (m, 14H), 7.00 (d, *J* = 2.23 Hz, 1H), 7.03 (d, *J* = 2.21 Hz, 2H), 7.05 (d, *J* = 2.30 Hz, 2H), 7.08-7.13 (m, 6H), 7.27 (s, 1H), 7.29 (s, 1H); ^{13}C NMR (100 MHz, $CDCl_3$) δ (ppm) 168.80, 168.63, 146.60, 146.40, 130.08, 130.04, 130.00, 116.09, 116.03, 115.87, 115.82, 98.28, 98.23, 95.42, 86.59, 82.53, 82.17, 72.67, 72.52, 69.51; 68,23, 68.98, 68.02, 66.46, 66.35, 56.47, 56.32, 55.95, 55.91, 42.63, 42.42, 41.66, 41.57, 37.81, 37.11, 36.69, 36.22, 35.01, 33.21, 32.27, 31.88, 30.12, 29.81, 29.64, 29.30, 29.23, 29.16, 26.57, 26.47, 23.51, 23.48; 22.64, 21.76, 18.13, 14.06, 9.27, 9.12; HRMS (m/z): $[M+Na]^+$ 909.4406 (calculated 909.4407) calculated for $C_{48}H_{67}FNaO_{14}^+$.

21-*p*-Dimethylaminobenzylidene digoxin (**BD-4**)

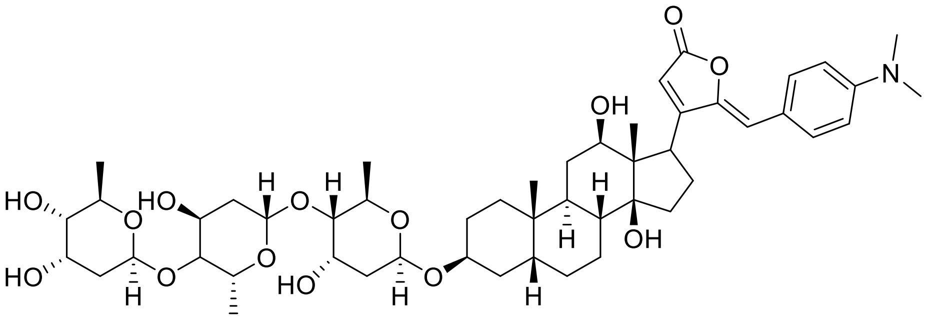

Yellow solid 0.57 g (0.63 mmol), yield 63%; m.p. 176-180 °C; IR (KBr) υ_{max}/cm^{-1} 3443, 2932, 1742, 1697, 1601, 1533, 1450, 1352, 1275, 1165, 1067, 1013, 868, 829; ^{1}H NMR (400 MHz, $CDCl_3$) δ (ppm) 0.76 (s, 3H), 0.91 (s, 3H), 1.18-1.31 (m, 9H), 1.59-2.29 (m, 25H), 3.01 (s, 6H), 3.08-3.33 (m, 7H), 3.42-3.53 (m, 3H), 3.72-3.87 (m, 4H), 4.03 (s, 1H), 4.25 (s, 2H), 4.82-4.95 (m, 4H), 6.08 (s, 1H), 6.55 (s, 1H), 6.66 (d, *J* = 8.87 Hz, 2H), 7.71 (d, *J* = 8.79 Hz, 2H); ^{13}C NMR (100 MHz, $CDCl_3$) δ (ppm) 171.35, 164.92, 150.57, 147.36, 132.51, 121.33,

113.00, 112.63, 11.88, 98.27, 98.21, 95.38, 86.44, 82.50, 82.12, 75.81, 72.60, 69.54, 68.22, 68.04, 67.96, 66.40, 66.28, 55.36, 42.12, 41.49, 40.07, 37.79, 37.08, 36.66, 36.23, 34.95, 32.83, 32.57, 32.47, 30.88, 30.61, 30.26, 30.17, 29.74, 29.64, 29.52, 26.46, 23.51, 21.62, 18.13, 8.66; HRMS (m/z): $[M+H]^+$ 912.5102 (calculated 912.5104) calculated for $C_{50}H_{74}NO_{14}^+$.

21-*m*-Nitrobenzylidene digoxin (**BD-5**)

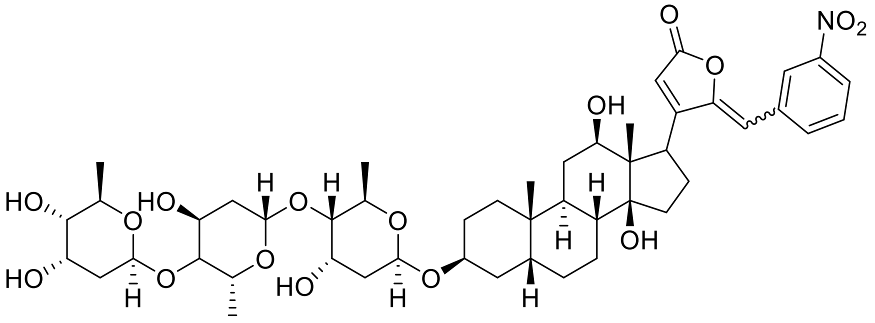

White solid, 0.52 g (0.57 mmol), yield 95%; m.p. 162-168 °C; IR (KBr) υ_{max}/cm^{-1} 3462, 2934, 2878, 1741, 1697, 1601, 1533, 1450, 1352, 1275, 1165, 1066, 1013, 868; ^1H NMR (400 MHz, $CDCl_3$) δ (ppm) 0.72 (s, 3H), 0.76 (s, 3H), 0.91 (d, *J* = 5.85 Hz, 7H), 1.22 (d, *J* = 6.16 Hz, 16H), 1.27 (d, *J* = 6.0 Hz, 10H), 1.48 (s, 6H), 1.59-2.22 (m, 59H), 2.71 (s, 3H), 3.05 (s, 2H), 3.11 (s, 2H), 3.19 (d, *J* = 3.19 Hz, 1H), 3.22 (dd, *J* = 2.83 Hz, *J* = 6.22 Hz, 3H), 3.25 (d, *J* = 2.85 Hz, 1H), 3.45 (dd, *J* = 7.47 Hz, *J* = 15.25 Hz, 3H), 3.53-3.60 (m, 3H), 3.71-3.87 (m, 11H), 4.02 (s, 2H), 4.17 (s, 1H), 4.22-4.27 (m, 5H), 4.83-4.93 (m, 8H), 7.34 (s, 1H), 7.38 (s, 1H), 7.48-7.57 (m, 5H), 8.00-8.04 (m, 2H), 8.16-8.19 (m, 2H); ^{13}C NMR (100 MHz, $CDCl_3$) δ (ppm) 202.48, 202.23, 198.63, 198.37, 169.52, 169.28, 148.39, 147.03, 146.53, 134.93, 134.64, 134.21, 133.81, 129.92, 129.83, 123.45, 122.93, 98.25, 95.37, 86.63, 82.50, 82.14, 72.60, 72.46, 69.48, 68.21, 68.03, 67.99, 66.41, 66.30, 56.60, 56.46, 55.95, 55.86; 42.36, 42.25, 41.48, 37.77, 37.07, 36.65, 36.16, 34.97, 33.19, 32.24, 30.08, 29.74, 29.22, 28.20,

27.72, 26.53, 26.42, 25.54, 23.47, 21.71, 18.12, 9.25, 9.14; HRMS (m/z): $[M+Na]^+$ 936.4348 (calculated 936.4358) calculated for $C_{48}H_{67}NNaO_{16}^+$.

21-*p*-Butoxy-*m*-methoxybenzylidene digoxin (**BD-7**)

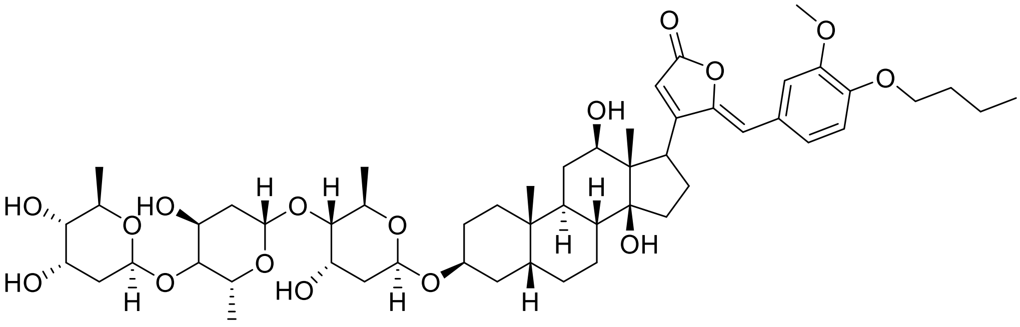

Yellow solid, 0.37 g (0.38 mmol), yield 64%; m.p. 118-124 °C; IR (KBr) υ_{max}/cm^{-1} 3481, 2932, 2876, 1738, 1695, 1601, 1450, 1367, 1256, 1163, 1068, 868; ^{1}H NMR (400 MHz, DMSO) δ (ppm) 0.65 (s, 3H), 0,85 (s, 3H), 0.94 (t, *J* = 7.3 Hz), 1.08-1.14 (m, 9H), 1.34-1.88 (m, 30H), 3.00 (t, *J* = 9.1 Hz, 2H), 3.13 (d, *J* = 9.1 Hz, 3H), 3.56-3.76 (m, 7H), 3.79 (s, 3H), 3.84-3.93 (m, 3H), 4.21 (s, 1H), 4.27 (s, 1H), 4.61-4.65 (m, 3H), 4.81 (d, *J* = 9.8 Hz, 3H), 5,00 (d, *J* = 5.2 Hz, 1H), 6.13 (s, 1H, H-22), 6.69 (s, 1H), 7.05 (d, *J* = 8.5 Hz, 1H), 7.23 (d, *J* = 8.5 Hz, 1H), 7.29-7.32 (m, 1H); ^{13}C NMR (100 MHz, DMSO) δ (ppm) 169.33, 166.35, 149.09, 148.74, 148.26, 125.80, 124.12, 113.60, 113.28, 112.82, 110.53, 98.94, 98.84, 95.21, 84.85, 81.80, 81.54, 73.63, 72.56, 71.99, 68.96, 67.75, 67.51, 67.39, 66.91, 66.16, 66.02, 55.62, 55.41, 41.85, 40.36, 38.31, 38.20, 37.79, 36.23, 34.60, 34.55, 32.29, 31.61, 30.64, 30.33, 30.08; 29.89, 29.52, 29.04, 26.38, 25.94, 23.56, 21.30, 18.65, 18.25, 17.92, 13.61, 9.3; HRMS (m/z): $[M+Na]^+$ 993,5183 (calculated 993,5182) calculated for $C_{53}H_{78}NaO_{16}^+$.

21-*m*-Butoxybenzylidene digoxin (**BD-8**)

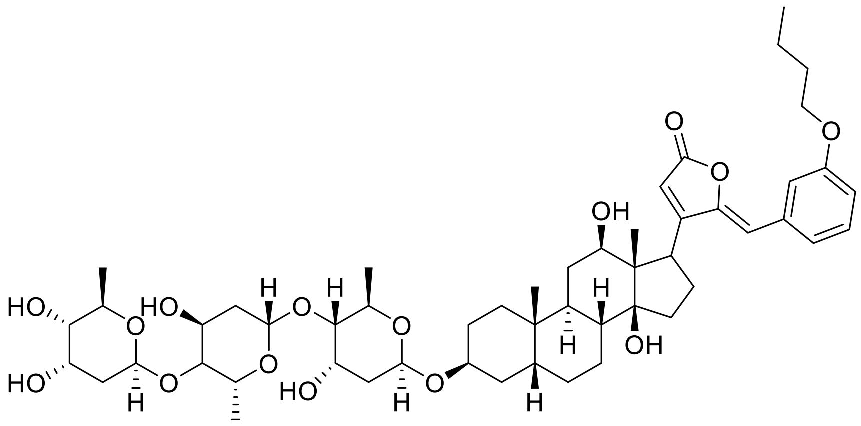

White solid, 0.45 g (0.48 mmol), yield 80%; m.p. 128-134 °C; IR (KBr) υ_{max}/cm^{-1} 3481, 2934, 2876, 1740, 1697, 1601, 1448, 1379, 1244, 1068, 868, 694, 729; ^{1}H NMR (400 MHz, DMSO) δ (ppm) 0.65 (s, 3H), 0.85 (s, 3H), 0.94 (t, *J* = 7.3 Hz, 3H), 1.08-1.14 (m, 9H), 1.32-1.89 (m, 30H), 2.96-3.03 (m, 2H), 3.13 (d, *J* = 8.8 Hz, 3H), 3.60-3.75 (m, 5H), 3.84-3.93 (m, 4H), 3.98 (t, *J* = 6,1 Hz, 1H), 4.21 (s, 1H), 4.27 (s, 1H), 4.61-4.65 (m, 2H), 4.82 (d, *J* = 9.5 Hz, 3H), 5.00 (d, *J* = 4.6 Hz, 1H), 6.20 (s, 1H), 6.73 (s, 1H), 6.93-6.98 (m, 1H), 7.20-7.25 (m, 2H), 7.32-7.44 (m, 1H); ^{13}C NMR (100 MHz, DMSO) δ (ppm) 169.14, 166.49, 158.74, 149,86, 134,28, 129.86, 129.66, 122.55, 115.89, 114.79, 114.71, 110.05, 98.95, 98.85, 95.22, 84.93, 81.81, 81.55, 73.68, 73.61, 72.57, 72.00, 68.96, 67.52, 67.39, 67.07, 66.92, 66.17, 66.03, 56.54, 55.68, 41.87, 38.32, 38.20, 37.80, 36.24, 34.60, 31.61, 30.64, 30.07, 29.87, 29.52, 28.90, 26.39, 25.94, 23.57, 21.29, 18.67, 18.25, 17.93, 13.61, 9.32; HRMS (m/z): $[M+Na]^+$ 963,5078 (calculated 963.5076) calculated for $C_{52}H_{76}NaO_{15}^+$.

21-*p*-Butoxybenzylidene digoxin (**BD-9**)

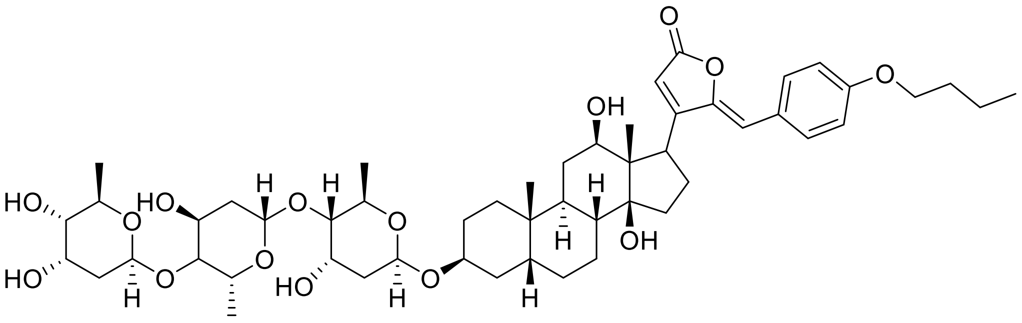

White solid, 0.77 g (0.82 mmol), yield 82%; m.p. 120-126 °C; IR (KBr) υ_{max}/cm^{-1} 3462, 2932, 2876, 1736, 1603, 1448, 1379, 1174, 1068, 870; ^{1}H NMR (400 MHz, DMSO) δ (ppm) 0.65 (s, 3H), 0.85 (s, 3H), 0.94 (t, *J* = 7.1 Hz, 3H), 1.07-1.16 (m, 9H), 1.31-1.88 (m, 30H), 3.00 (d, *J* = 8.7 Hz, 2H), 3.13 (d, *J* = 8.7 Hz, 3H), 3.59-3.75 (m, 7H), 3.84-3.94 (m, 3H), 4.16-4.29 (m, 4H), 4.82 (d, *J* = 9.5 Hz, 3H), 6.13 (s, 1H), 6.70 (s, 1H), 7.01 (d, *J* = 8.2 Hz, 2H), 7.63 (d, *J* = 8.2 Hz, 2H); ^{13}C NMR (100 MHz, DMSO) δ (ppm) 169.34, 166.34, 159.09, 148.22, 131.78, 125.60, 114.82, 113.61, 110.15, 98.92, 98.81, 95.19, 84.85, 81.79, 81.52, 73.59, 72.62, 71.95, 68.97, 67.50, 67.36, 67.17, 66.89, 66.14, 66.00, 55.60, 41.80, 40.34, 38.28, 38.20, 37.76, 36.22, 34.58, 32.27, 31.61, 30.58, 30.05, 29.87, 29.51, 28.98, 26.39, 25.94, 25.02, 23.55, 21.28, 18.61, 18.22, 17.90, 13.58, 9.27; HRMS (m/z): $[M+Na]^{+}$ 963,5079 (calculated 963,5076) calculated for $C_{52}H_{76}NaO_{15}^{+}$.

21-*o*-Butoxybenzylidene digoxin (**BD-10**)

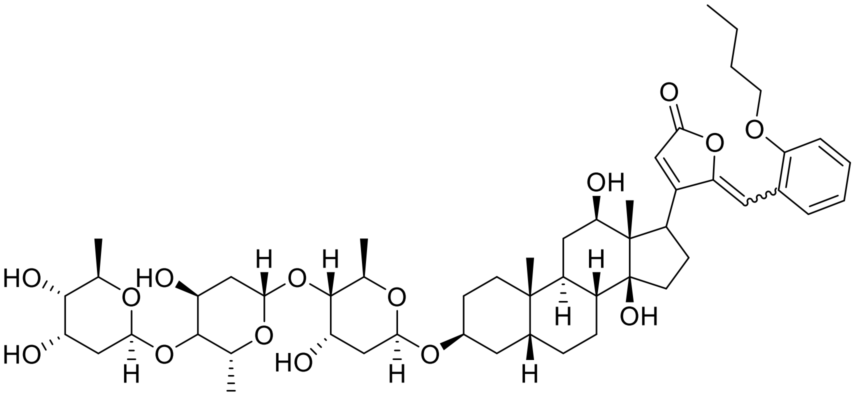

White solid, 0.33 g (0.35 mmol), yield 58%; m.p. 130-136 °C; IR (KBr) υ_{max}/cm^{-1} 3449, 2932, 2876, 1740, 1697, 1601, 1452, 1381, 1163, 1068, 870, 752; ^{1}H NMR (400 MHz, $CDCl_3$) δ (ppm) 0.49 (s, 3H), 0.75 (s, 3H), 0.76-0.77 (m, 6H), 0.87-0.90 (m, 14H), 0.91-0.92 (m, 15H), 0.94-1.02 (m, 34H), 1.20-1.24 (m, 67H), 1.42-1.84 (m, 223H), 2.06-2.15 (m, 32H), 2.95-2.96 (m, 1H), 2.99-3.08 (m, 16H), 3.17-3.29 (m, 36H), 3.72-4.4 (m, 67H), 4.22-4.26 (m, 18H), 4.83-4.92 (m, 28H), 6.55 (s, 1H); 6.75-6.79 (m, 5H), 6.81 (s, 1H), 6.81-6.85 (m, 16H), 7.10-7.11 (m, 1H), 7.14-7.19 (m, 8H), 7.30-7.32 (m, 2H); ^{13}C NMR (100 MHz, $CDCl_3$) δ (ppm) 172.92, 168.82, 165.73, 147.26, 147.05, 140.82, 132.72, 132.62, 129.90, 129.72, 122.06, 120.88, 111.48, 111.17, 110.94, 107.41, 98.28, 98.23, 95.42, 86.68, 86.61, 86.25, 85.99, 82.55, 82.17, 72.66, 72.57, 69.52, 68.24, 68.07, 68.01, 67.75, 67.69, 67.63, 66.46, 66.34, 56.27, 56.15, 55.54, 54.51, 51.72, 51.29, 48.47, 43.19, 43.15, 42.42, 41.80, 41.64, 41.56, 37.81, 37.12, 37.01, 36.70, 36.24, 35.11, 35.01, 34.95, 33.04, 32.92, 32.32, 32.24, 32.19, 31.47, 31.31, 31.28, 31.22, 31.08, 30.30, 30.13, 29.82, 29.65, 28.91, 27.45, 26.59, 26.49, 26.37, 25.90, 23.54, 23.39, 21.74, 19.39, 19.34, 19.29, 19.15, 19.06, 18.95, 18.13, 17.83, 17.77, 14.06, 13.78, 9.11, 8.96; HRMS (m/z): $[M+Na]^+$ 963.5076 (calculated 963.5076) calculated for $C_{52}H_{76}NaO_{15}^+$.

21-*p*-Hexoxybenzylidene digoxin (**BD-13**)

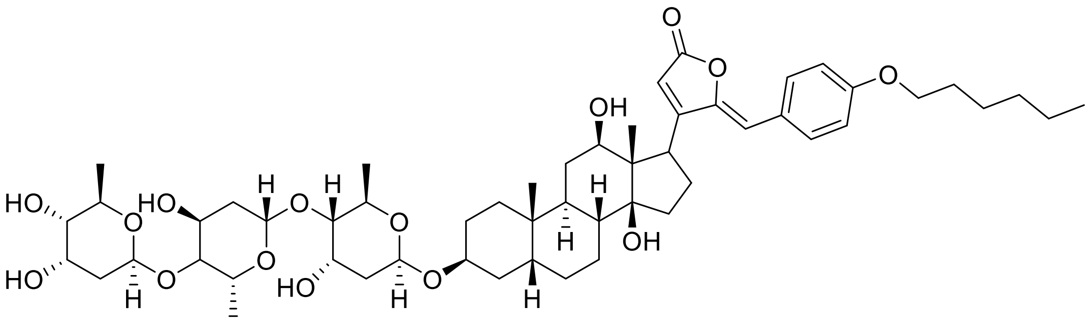

Yellow solid, 0.33 g (0.34 mmol), yield 57%; m.p. 90-94 °C; IR (KBr) υ_{max}/cm^{-1} 3445, 2926, 2855, 1740, 1695, 1603, 1456, 1379, 1165, 1068, 870; ^{1}H NMR (400 MHz, DMSO) δ (ppm) 0.83-0.95 (m, 9H), 1.08-1.14 (m, 9H), 1.21-1.89 (m, 30H), 2.18 (s, 1H), 2.96-3.03 (m, 1H), 3.13 (d, *J* = 8.9 Hz, 2H), 3.58-3.74 (m, 7H), 3.74-3.92 (m, 3H), 4.18-4.28 (m, 4H), 4.51-4.59 (m, 4H), 4.82 (d, *J* = 9.4 Hz, 3H), 6.61 (s, 1H), 6.87 (s, 1H), 7.63-7.76 (m, 4H); ^{13}C NMR (100 MHz, DMSO) δ (ppm) 169.90, 166.74, 149.71, 139.01, 131.36, 128.50, 127.86, 124.74, 98.86, 98.76, 97.78, 95.17, 91.94, 81.73, 81.47, 72.52, 71.94, 68.93, 67.48, 67.34, 67.22, 66.87, 66.44, 66.10, 65.97, 63.62, 61.13, 55.79, 38.26, 38.16, 37.73, 36.54, 34.24, 30.29, 30.22, 29.06, 28.86, 28.54, 28.45, 28.22, 27.08, 25.48, 24.95, 24.41, 24.24, 22.27, 22.12, 21.92, 20.89, 10.09, 18.73, 18.19, 17.88, 13.75; MS (m/z): [M-H]$^{+}$ 967.2 (calculated 967.54) calculated for $C_{54}H_{79}O_{15}^{-}$.

21-*m*-Hexoxybenzylidene digoxin (**BD-14**)

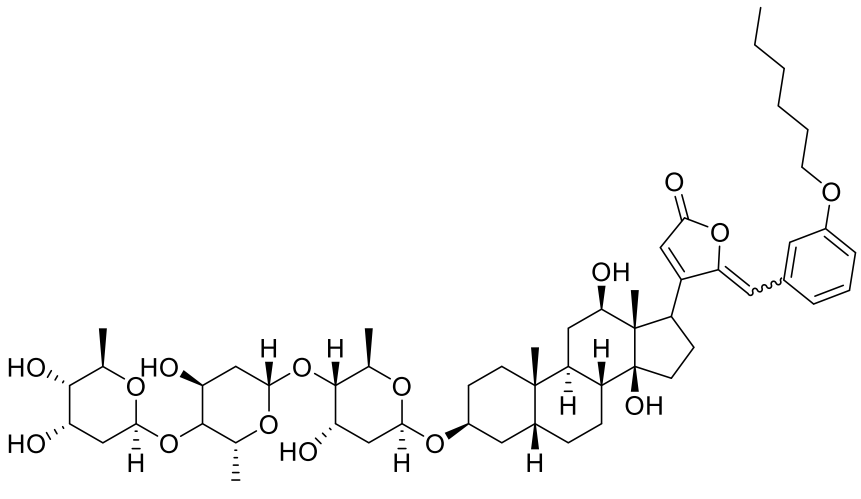

White solid, 0.44 g (0.45 mmol), yield 75%; m.p. 118-122 °C; IR (KBr) υ_{max}/cm^{-1} 3466, 2932, 2876, 1740, 1695, 1601, 1448, 1379, 1244, 1068, 870, 727, 698; ^{1}H NMR (400 MHz, $CDCl_3$) δ (ppm) 0.72 (s, 3H), 0.74 (s, 3H), 0.89-0.92 (m, 12H), 1.21-1.28 (m, 33H), 1.30-2.18 (m, 96H), 2.98-3.09 (m, 4H), 3.19 (d, *J* = 2.9 Hz, 1H), 3.22 (dd, *J* = 6.3 Hz, *J* = 2.9 Hz, 2H),

3.26 (dd, *J* =6.3 Hz, *J* = 2.9 Hz, 2H), 3.29 (d, *J* = 2.9 Hz, 1H), 3.39 (t, *J* = 8.4 Hz, 2H), 3.52-3.59 (m, 2H), 3.71-3.85 (m, 8H), 3.88-3.93 (m, 6H), 3.96 (s, 1H), 4.02 (s, 2H), 4.21-4.26 (m, 5H), 4.84 (d, *J* = 1.7 Hz, 1H), 4.87 (d, *J* = 1.7 Hz, 1H), 4.87-4.89 (m, 2H), 4.89-4.92 (m, 2H), 6.62-6.69 (m, 5H), 6.79-6.81 (m, 1H), 6.81-6.83 (m, 1H), 7.18-7.24 (m, 2H), 7.28-7.29 (m, 1H); ^{13}C NMR (100 MHz, $CDCl_3$) δ (ppm) 168.83, 168.63, 159.51, 146.77, 146.57, 133.70, 133.48, 129.89, 120.36, 114.98, 114.86, 114.03, 113.80, 98.28, 98.23, 95.43, 86.59, 82.55, 82.19, 72.68, 72.54, 69.50, 68.24, 68.08, 68.04, 67.98, 66.46, 66.35, 56.92, 56.79, 56.41, 56.33, 42.75, 42.50, 41.71, 41.64, 37.82, 37.13, 36.71, 36.24, 35.02, 33.20, 32.27, 31.53; 30.14, 29.84, 29.65, 29.15, 28.07, 27.66, 26,59, 26.49, 25.67, 23.53, 23.49, 22.56, 21.78, 18.14, 13.99, 9.28, 9.10; MS (m/z): $[M-H]^+$ 967.2 (calculated 967.54) calculated for $C_{54}H_{79}O_{15}^-$.

21-*p*-Hexoxy-*m*-methoxybenzylidene digoxin (**BD-15**)

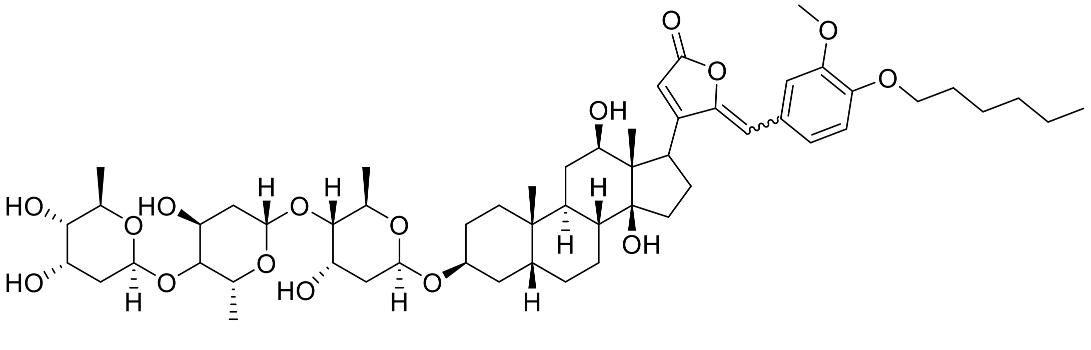

White solid, 0.31 g (0.31 mmol), yield 52%; m.p. 88-92 °C; IR (KBr) υ_{max}/cm^{-1} 3474, 2930, 2874, 1734, 1695, 1599, 1450, 1379, 1256, 1163, 1070, 868; ^{1}H NMR (400 MHz, $CDCl_3$) δ (ppm) 0.73 (s, 3H), 0.75 (s, 3H), 0.87-0.93 (m, 26H), 0.94-1.00 (m, 14H), 1.21-1.24 (m, 25H), 1.59-2.20 (m, 102H), 3.18-3.30 (m, 12H), 3.74-3.79 (m, 7H), 3.80 (s, 3H), 3.83 (s, 3H), 3.89-3.90 (m, 1H), 3.94 (s, 1H), 3.97 (t, *J* = 6.9 Hz, 6H), 4.00-4.03 (m, 3H), 4.22-4.26 (m, 7H), 4.33 (t, *J* = 6.9, 3H); 4.84 (d, *J* = 1.6 Hz, 1H), 4.87 (d, *J* = 1.6 Hz, 1H), 4.88 (dd, *J* = 3.7 Hz, *J*

= 1.6 Hz, 3H), 4.91 (dd, *J* = 3.7 Hz, *J* = 1.6 Hz, 3H), 6.60-6.61 (m, 1H), 6.62 (s, 1H), 6.62-6.66 (m, 3H), 6.80 (d, *J* = 1.6 Hz, 1H), 6.82 (d, *J* = 1.6 Hz, 1H), 6.97 (s, 1H), 7.51-7.54 (m, 2H), 7.69-7.72 (m, 1H); ^{13}C NMR (100 MHz, $CDCl_3$) δ (ppm) 168.68, 168.34, 149.76, 148.44, 146.57, 146.40, 130.87, 128.80, 125.47, 120.79, 120.60, 113.29, 112.34, 111.97, 98.27, 98.22, 95.43, 86.57, 82.55, 82.19, 72.70, 72.53, 69.50, 69.11, 68.24, 68.06, 66.46, 66.36, 64.35, 56.39, 56.30, 56.09, 55.99, 42.84, 42.50, 41.73, 41.64, 37.82, 37.20, 37.13, 36.71, 36.24, 35.03, 33.23, 32.27, 31.89, 31.54, 30.31, 30.15, 29.84, 29.66, 29.45, 29.32, 29.24, 29.14, 29,05, 28.97, 28.04, 26.60, 26.49, 25.58, 23.54, 22.51, 22.44, 21.78, 18.14, 14.07, 13.98, 9.28, 9.11; MS (m/z): $[M+H]^+$ 969.2 (calculated 999.56) calculated for $C_{55}H_{83}O_{16}^+$.

21-*o*-Hexoxybenzylidene digoxin (**BD-16**)

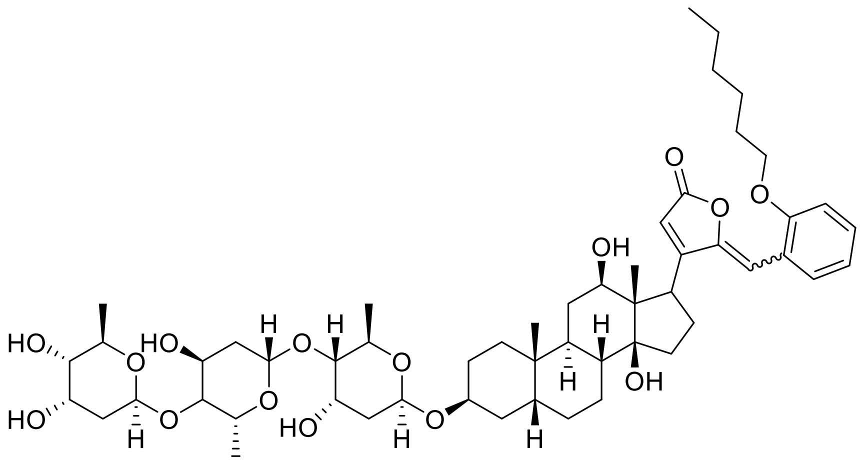

White solid, 0.33 g (0.34 mmol), yield 57%; m.p. 130-136 °C; IR (KBr) υ_{max}/cm^{-1} 3474, 2932, 2874, 1742, 1697, 1602, 1454, 1379, 1068, 870, 752; ^{1}H NMR (400 MHz, $CDCl_3$) δ (ppm) 0.75 (s, 3H), 0.77 (s, 1H), 0.87 (s, 1H), 0.89 (s, 3H), 0.90-0.92 (m, 8H), 1.20-1.29 (m, 42H), 1.71-2.16 (m, 80H), 3.17-3.29 (m, 9H), 3.36 (t, *J* = 8.6 Hz, 2H), 3.73-3.89 (m, 13H), 3.92 (s, 1H), 4.00-4.02 (m, 2H), 4.07-4.10 (m, 3H), 4.22-4.26 (m, 5H), 4.84 (d, *J* = 1.8

Hz, 1H), 4.87 (d, *J* = 1.8 Hz, 1H), 4.87-4.89 (m, 3H), 4.89-4.92 (m, 3H), 6.76 (s, 1H), 6.78 (s, 1H), 6.91-6.96 (m, 2H), 7.02-7.05 (m, 1H), 7.09-7.10 (m, 1H), 7.13-7.15 (m, 1H), 7.15-7.17 (m, 1H), 7.23-7.24 (m, 1H), 7.27-7.29 (m, 1H); ^{13}C NMR (100 MHz, $CDCl_3$) δ (ppm) 165.69, 155.99, 145.58, 143.29, 132.68, 129.70, 121.23, 120.85, 120.79, 111.72, 111.18, 98.27, 98.22, 95.42, 86.68, 86.28, 82.55, 82.17, 72.66, 72.55, 69.51, 68.24, 68.07, 68.02, 66.46, 66.34, 56.25, 56.14, 43.73, 42.45, 41.81, 41.58, 37.81, 37.12, 36.70, 36.24, 35.02, 33.37, 33.05, 32.24, 31.47, 30.13, 29.83, 29.02, 28,96, 28.72, 27.76, 27.44, 26.60, 26.50, 25.54, 25.46, 23.53, 22.54, 21.86, 21.75, 18.14, 13.99, 9.28, 9.12; MS (m/z): $[M-H]^+$ 967.2 (calculated 967.54) calculated for $C_{54}H_{79}O_{15}^-$.

FIGURE LEGENDS

Figure S1: NKA activity (%) from Sf9 cell membrane preparations after 21-BD (A) and BD-1 (B) treatment for 20 min. * $p < 0.05$ differences between NKA α1β1 and α2β1 isoforms. Each point represents the mean ± SEM of at least three independent experiments performed in triplicate.

Figure S2: Ramachandran plot of rat NKA α1 isoform.

Figure S3: Ramachandran plot of rat NKA α2 isoform.

Figure S4: Ramachandran plot of rat NKA α3 isoform.

Figure S5: Alignment comparing the whole primary amino acid sequences from rat NKA α isoforms.

Figure S6: 21-BD molecular docking on rat NKA α1 (A), α2 (B), and α3 (C) isoforms. The green and magenta circles represent residues involved in van der Waals and polar interactions, respectively. The blue halo around the residue is proportional to the solvent accessible surface. Pi interactions are represented by an orange line and symbols indicating the specific interaction. The green dashed arrows are directed towards the electron donor and represent

hydrogen bonds with amino acid main chains. The blue dashed arrows are directed towards the electron donor and represent hydrogen bonds with amino acid side-chains.

Figure S7: BD-3 molecular docking on rat NKA α1 (A) and α3 (B) isoforms. The green and magenta circles represent residues involved in van der Waals and polar interactions, respectively. The blue halo around the residue is proportional to the solvent accessible surface. Pi interactions are represented by an orange line and symbols indicating the specific interaction. The green dashed arrows are directed towards the electron donor and represent hydrogen bonds with amino acid main chains. The blue dashed arrows are directed towards the electron donor and represent hydrogen bonds with amino acid side-chains.

Figure S8: BD-8 molecular docking on rat NKA α1 (A) and α3 (B) isoforms. The green and magenta circles represent residues involved in van der Waals and polar interactions, respectively. The blue halo around the residue is proportional to the solvent accessible surface. Pi interactions are represented by an orange line and symbols indicating the specific interaction. The green dashed arrows are directed towards the electron donor and represent hydrogen bonds with amino acid main chains. The blue dashed arrows are directed towards the electron donor and represent hydrogen bonds with amino acid side-chains.

Figure S9: BD-13 molecular docking on rat NKA α1 (A) and α3 (B) isoforms. The green and magenta circles represent residues involved in van der Waals and polar interactions, respectively. The blue halo around the residue is proportional to the solvent accessible surface. Pi interactions are represented by an orange line and symbols indicating the specific interaction. The green dashed arrows are directed towards the electron donor and represent

hydrogen bonds with amino acid main chains. The blue dashed arrows are directed towards the electron donor and represent hydrogen bonds with amino acid side-chains.

Figure S10: BD-14 molecular docking on rat NKA α1 (A) and α2 (B) isoforms. The green and magenta circles represent residues involved in van der Waals and polar interactions, respectively. The blue halo around the residue is proportional to the solvent accessible surface. Pi interactions are represented by an orange line and symbols indicating the specific interaction. The green dashed arrows are directed towards the electron donor and represent hydrogen bonds with amino acid main chains. The blue dashed arrows are directed towards the electron donor and represent hydrogen bonds with amino acid side-chains.

Figure S11: BD-15 molecular docking on rat NKA α1 (A) and α2 (B) isoforms. The green and magenta circles represent residues involved in van der Waals and polar interactions, respectively. The blue halo around the residue is proportional to the solvent accessible surface. Pi interactions are represented by an orange line and symbols indicating the specific interaction. The green dashed arrows are directed towards the electron donor and represent hydrogen bonds with amino acid main chains. The blue dashed arrows are directed towards the electron donor and represent hydrogen bonds with amino acid side-chains.

FIGURES

Figure S1

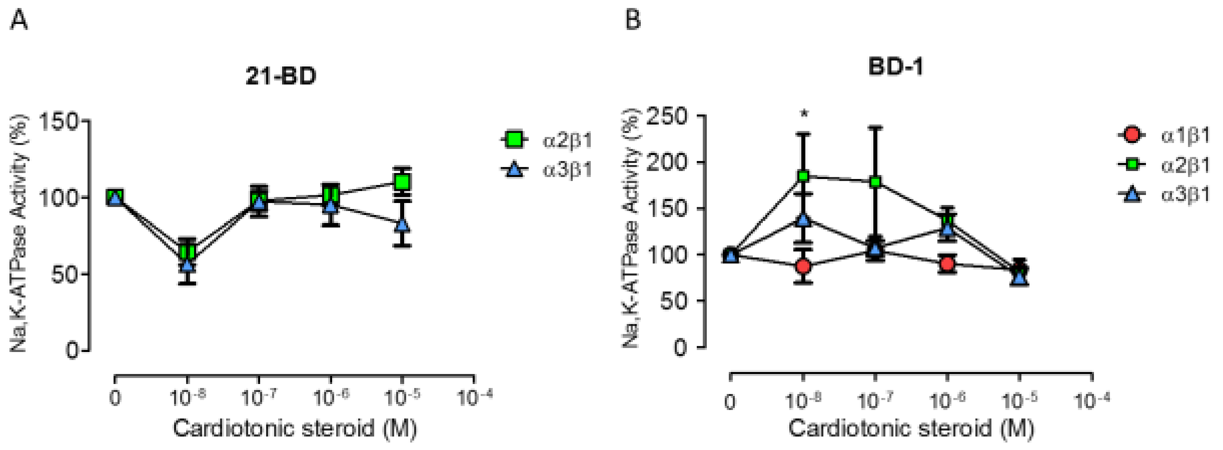

Figure S2

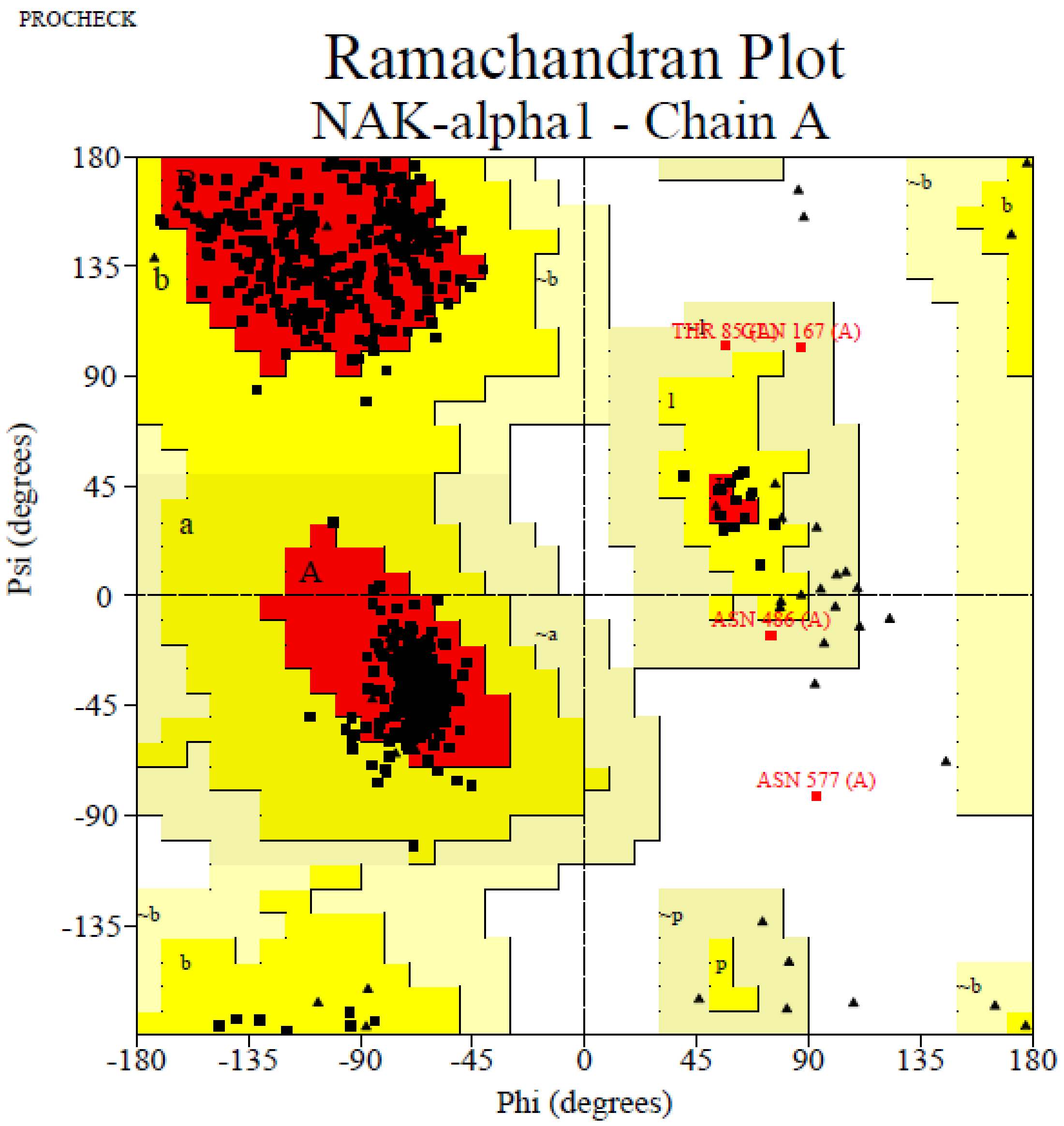
PROCHECK
Ramachandran Plot
NAK-alpha1 - Chain A
THR 85 (A)
GLN 167 (A)
ASN 486 (A)
ASN 577 (A)
Psi (degrees)
Phi (degrees)
180
135
90
45
0
-45
-90
-135
-180
-135
-90
-45
0
45
90
135
180
A
B
a
b
~a
~b
l
p
~p

Figure S3

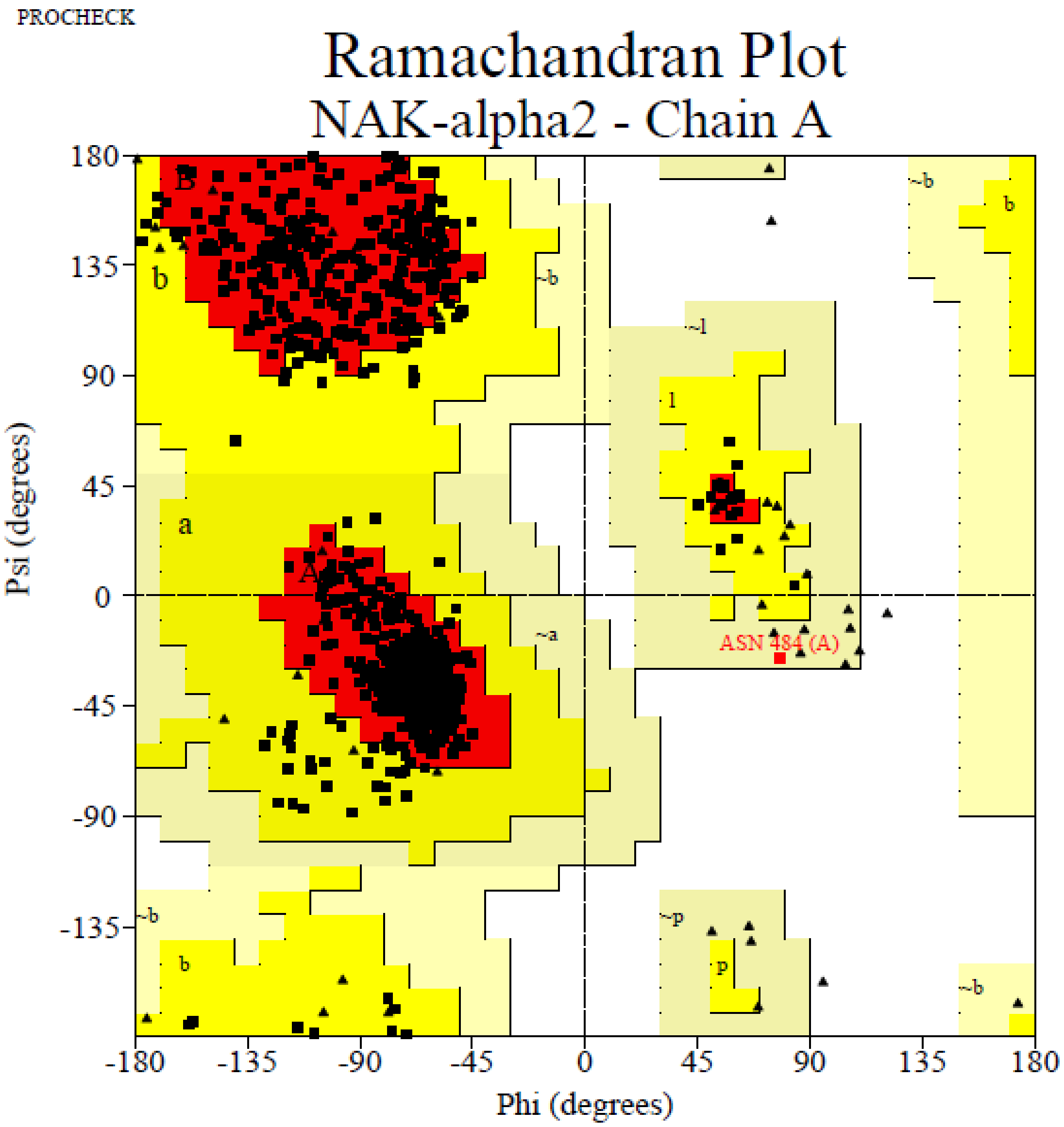
PROCHECK
Ramachandran Plot
NAK-alpha2 - Chain A
Psi (degrees)
Phi (degrees)
180
135
90
45
0
-45
-90
-135
-180
ASN 484 (A)

Figure S4

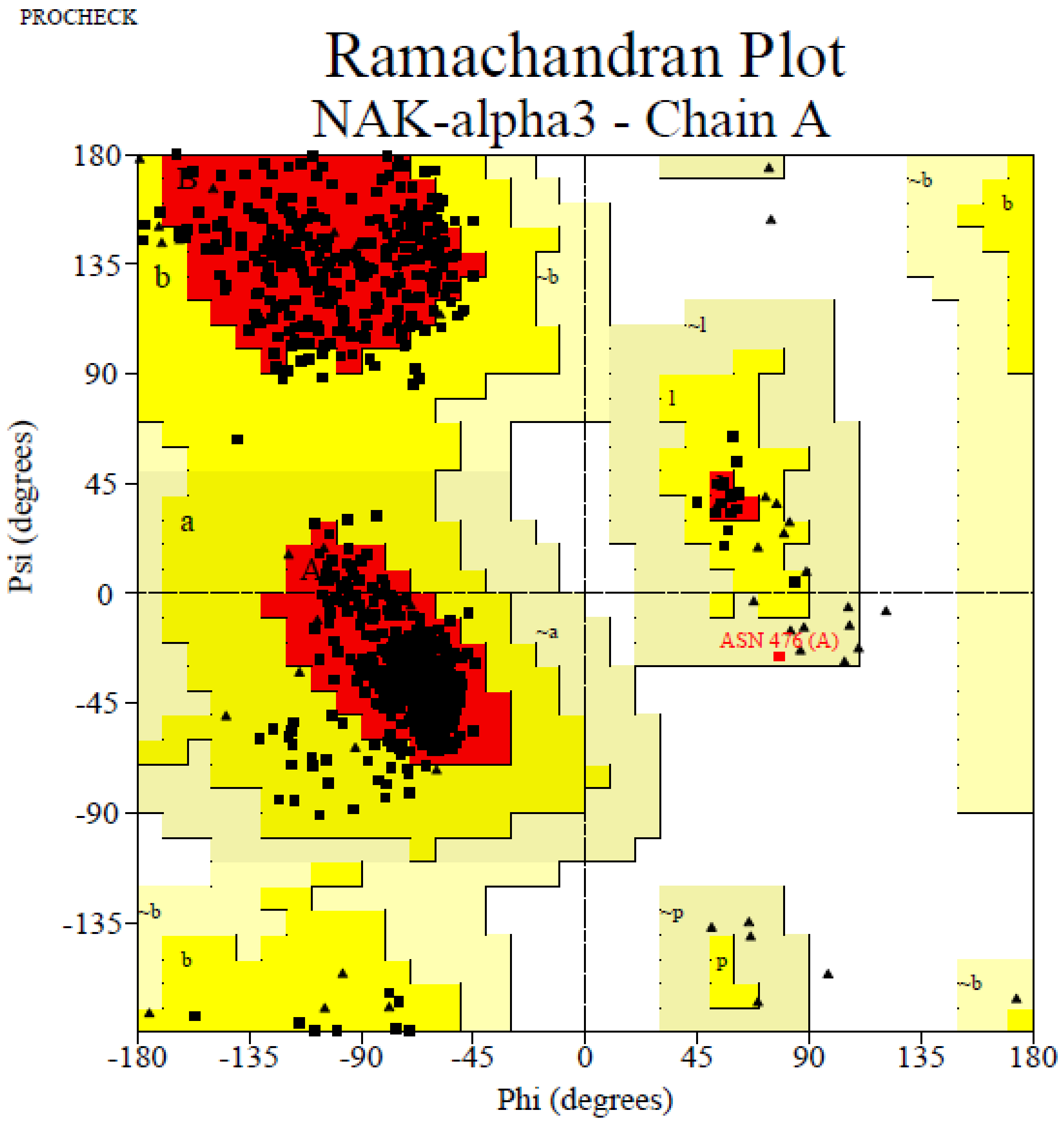
PROCHECK
Ramachandran Plot
NAK-alpha3 - Chain A
180
135
90
45
0
-45
-90
-135
Psi (degrees)
-180
-135
-90
-45
0
45
90
135
180
Phi (degrees)
B
b
~b
a
A
~a
~l
l
ASN 476 (A)
~p
p

Figure S5

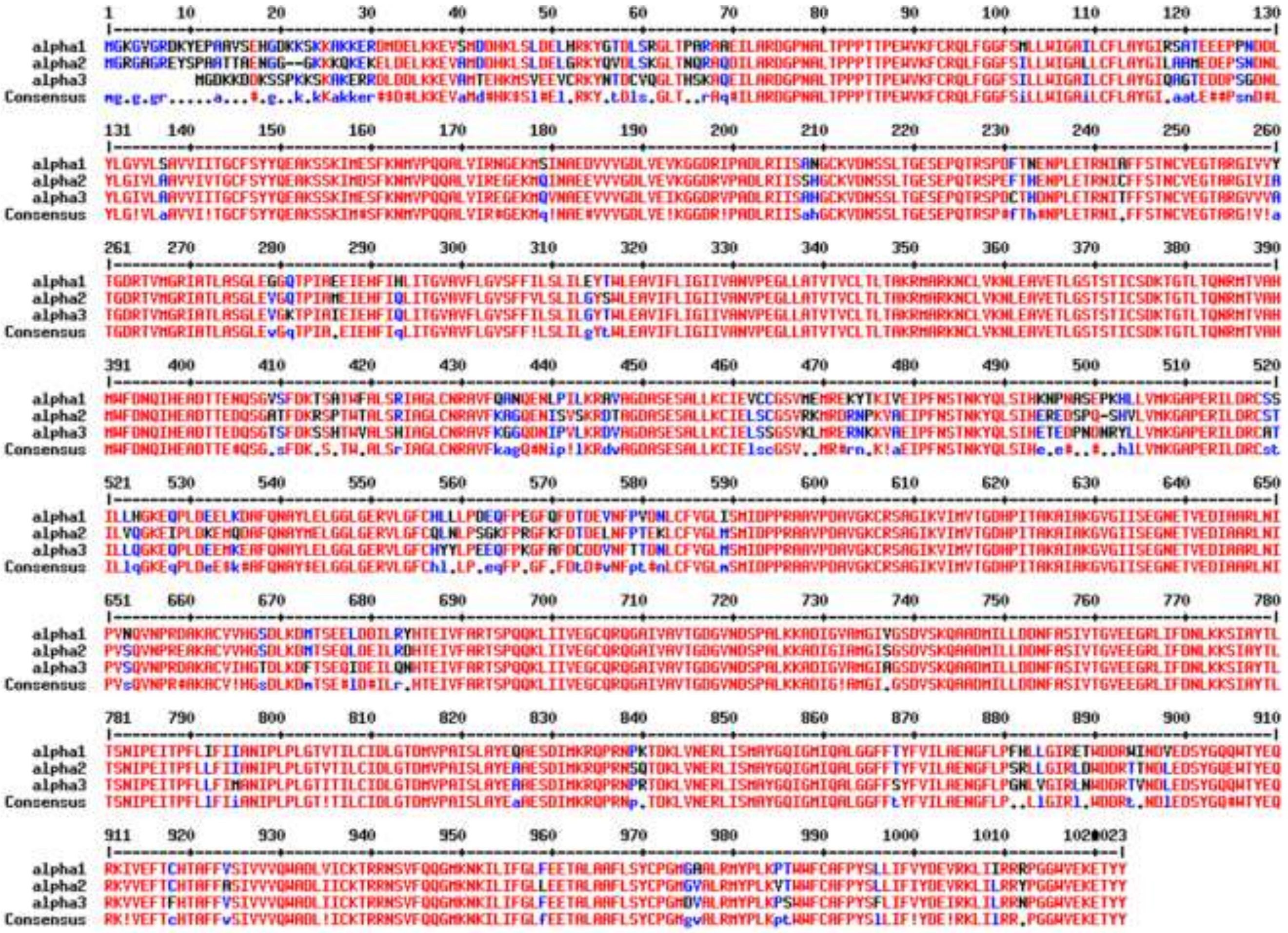

Figure S6

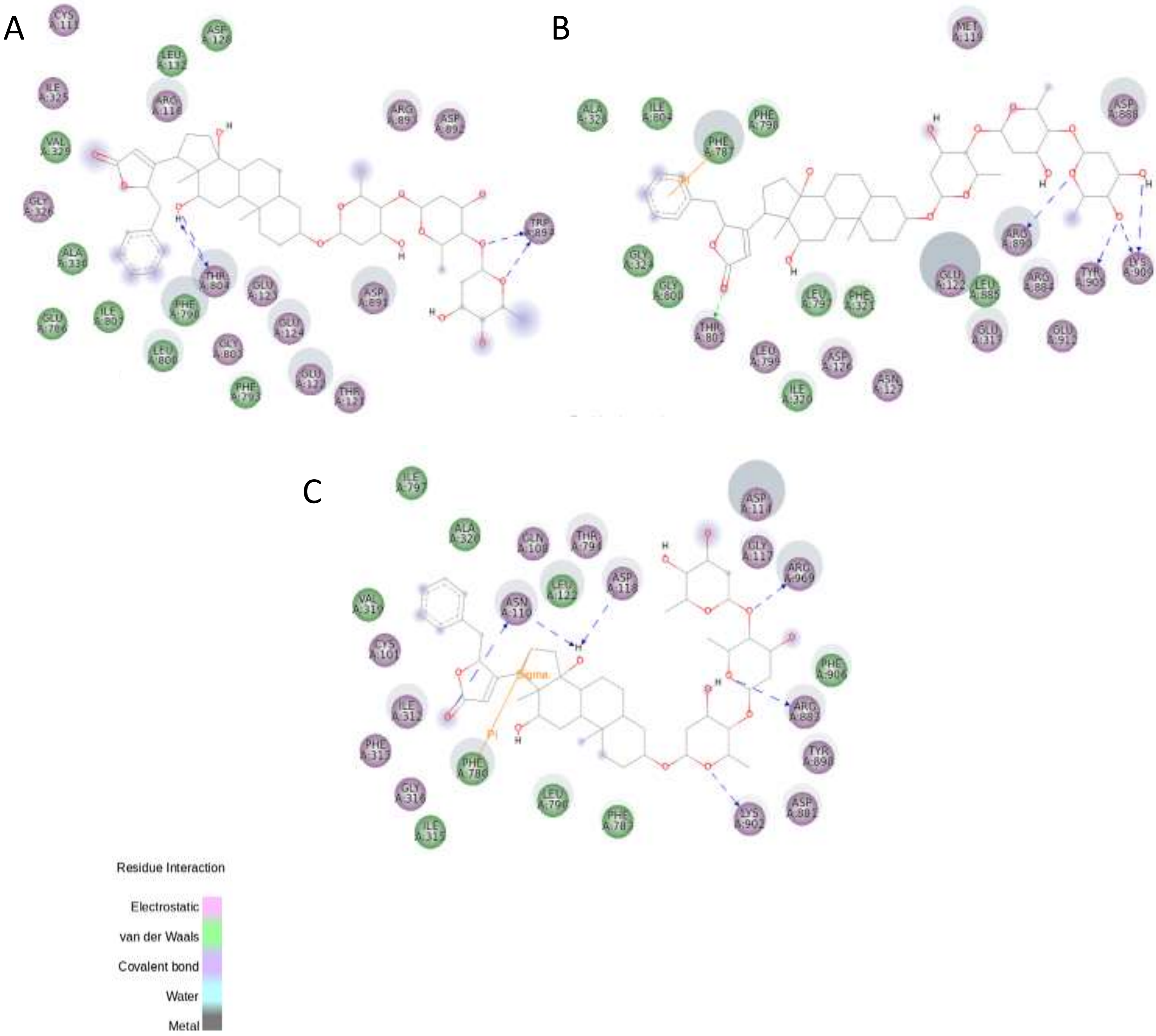
A
B
C
Residue Interaction
Electrostatic
van der Waals
Covalent bond
Water
Metal

Figure S7

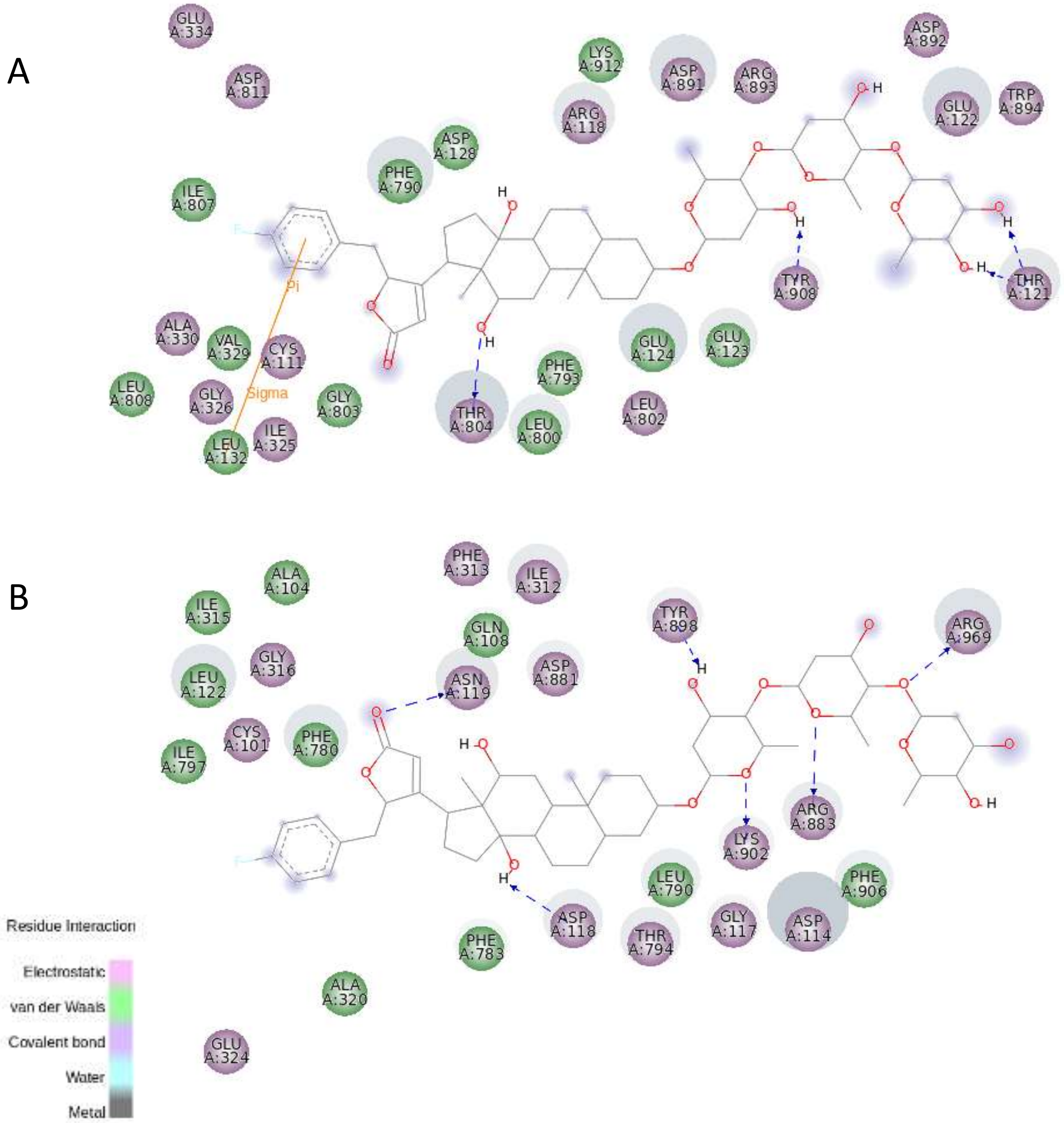
A
GLU A:334
ASP A:811
LYS A:912
ASP A:891
ARG A:893
ASP A:892
ARG A:118
GLU A:122
TRP A:894
ASP A:128
PHE A:790
ILE A:807
Pi
TYR A:908
THR A:121
ALA A:330
VAL A:329
CYS A:111
GLU A:124
GLU A:123
LEU A:808
GLY A:326
Sigma
GLY A:803
THR A:804
PHE A:793
LEU A:802
LEU A:800
LEU A:132
ILE A:325
B
PHE A:313
ILE A:312
ALA A:104
ILE A:315
TYR A:898
ARG A:969
GLN A:108
GLY A:316
LEU A:122
ASN A:119
ASP A:881
CYS A:101
PHE A:780
ILE A:797
ARG A:883
LYS A:902
LEU A:790
PHE A:906
ASP A:118
GLY A:117
ASP A:114
THR A:794
PHE A:783
ALA A:320
GLU A:324
Residue Interaction
Electrostatic
van der Waals
Covalent bond
Water
Metal

Figure S8

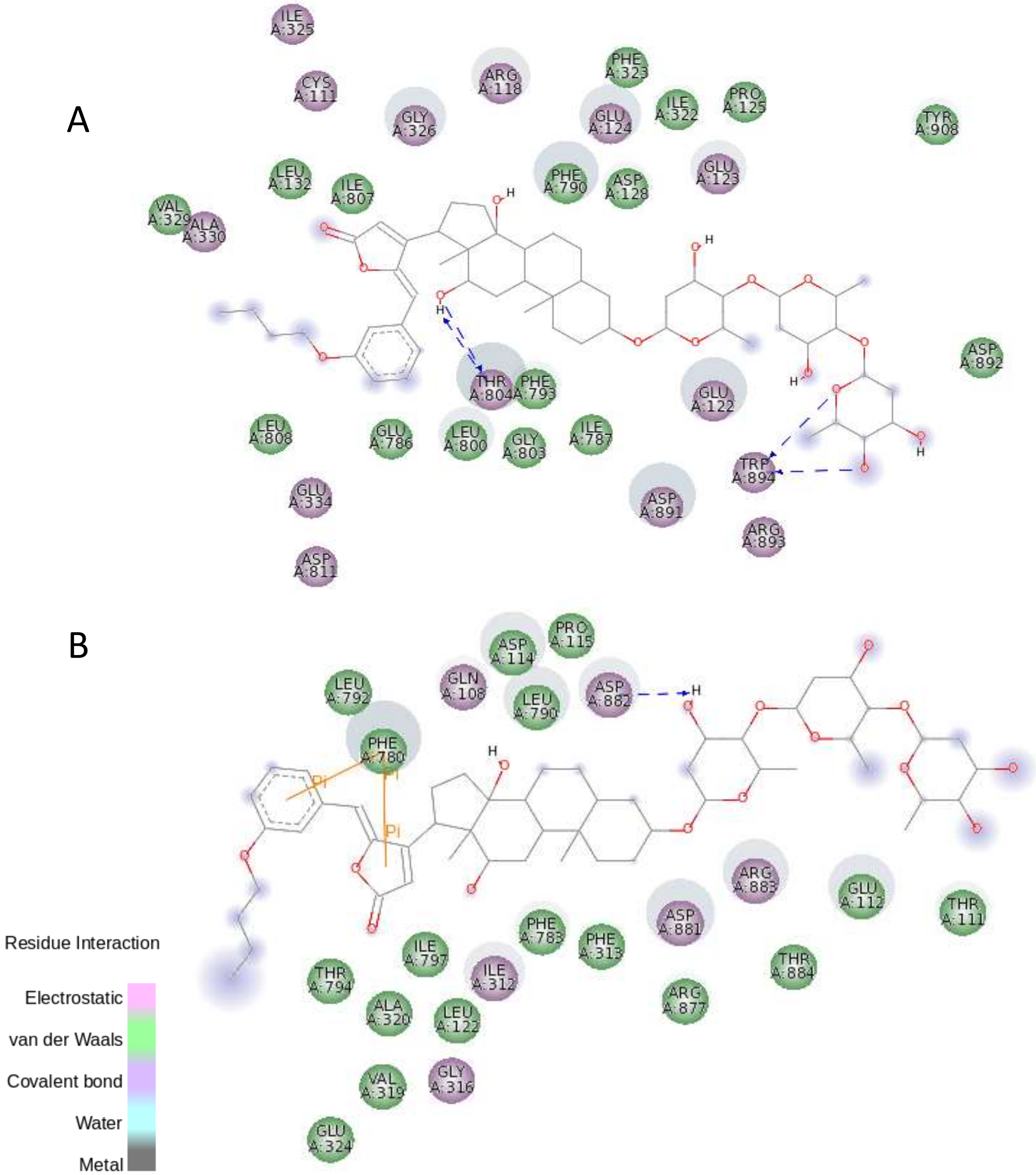
A
ILE A:325
CYS A:111
ARG A:118
GLY A:326
PHE A:323
GLU A:124
ILE A:322
PRO A:125
TYR A:908
LEU A:132
ILE A:807
PHE A:790
ASP A:128
GLU A:123
VAL A:329
ALA A:330
THR A:804
PHE A:793
GLU A:122
ASP A:892
LEU A:808
GLU A:786
LEU A:800
GLY A:803
ILE A:787
TRP A:894
GLU A:334
ASP A:891
ARG A:893
ASP A:811
B
PRO A:115
ASP A:114
LEU A:792
GLN A:108
LEU A:790
ASP A:882
PHE A:780
Pi
ARG A:883
GLU A:112
THR A:111
PHE A:783
PHE A:313
ASP A:881
ILE A:797
THR A:794
ILE A:312
THR A:884
ALA A:320
LEU A:122
ARG A:877
VAL A:319
GLY A:316
GLU A:324
Residue Interaction
Electrostatic
van der Waals
Covalent bond
Water
Metal

Figure S9

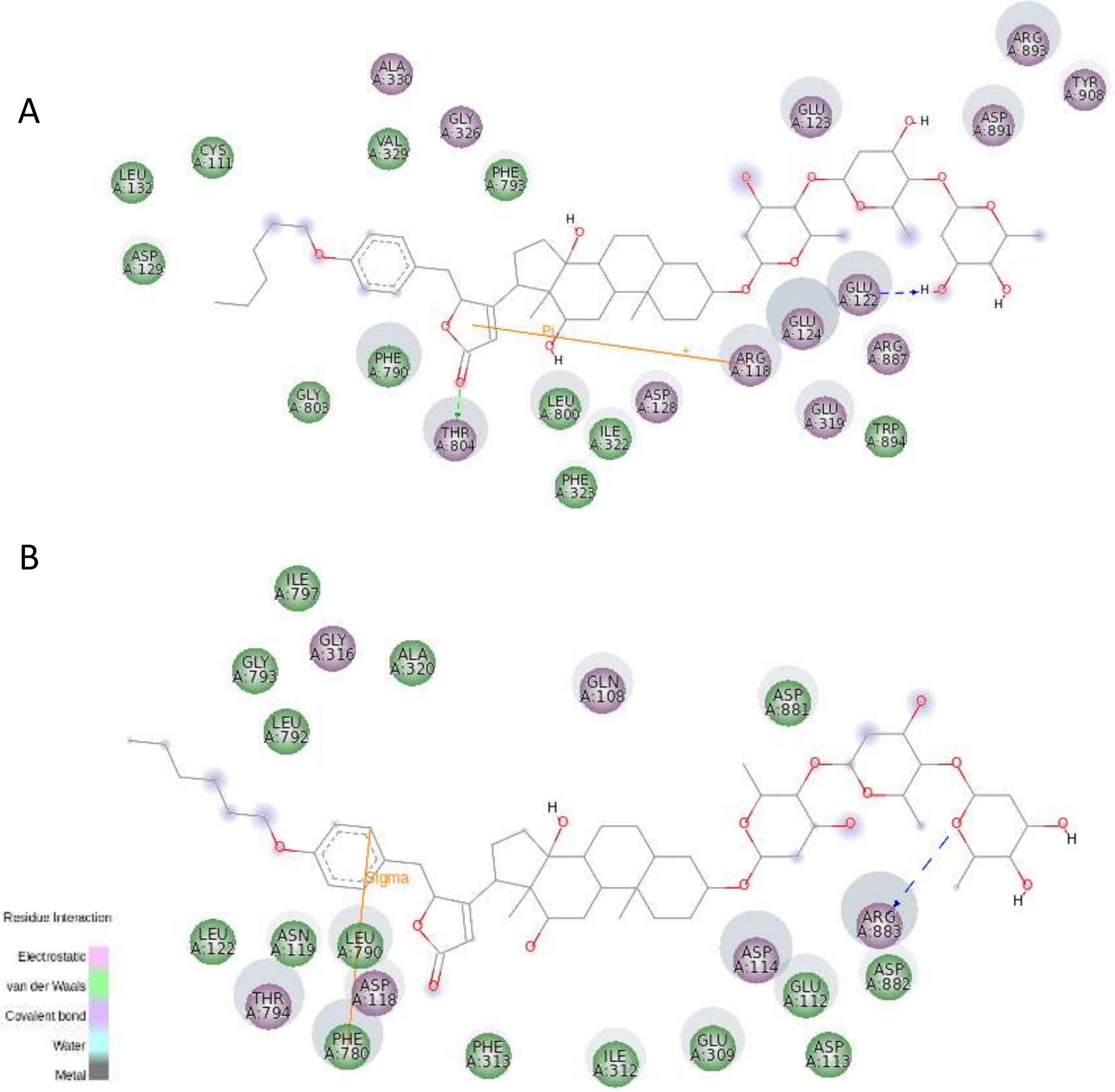
A
ALA A:330
GLY A:326
VAL A:329
CYS A:111
LEU A:132
PHE A:793
ASP A:129
GLU A:123
ARG A:893
TYR A:908
ASP A:891
GLU A:122
GLU A:124
ARG A:887
ARG A:118
Pi
PHE A:790
GLY A:803
THR A:804
LEU A:800
ASP A:128
ILE A:322
PHE A:323
GLU A:319
TRP A:894
B
ILE A:797
GLY A:316
ALA A:320
GLY A:793
LEU A:792
GLN A:108
ASP A:881
Sigma
LEU A:122
ASN A:119
LEU A:790
ASP A:118
THR A:794
PHE A:780
PHE A:313
ILE A:312
GLU A:309
ASP A:113
ASP A:114
GLU A:112
ARG A:883
ASP A:882
Residue Interaction
Electrostatic
van der Waals
Covalent bond
Water
Metal

Figure S10

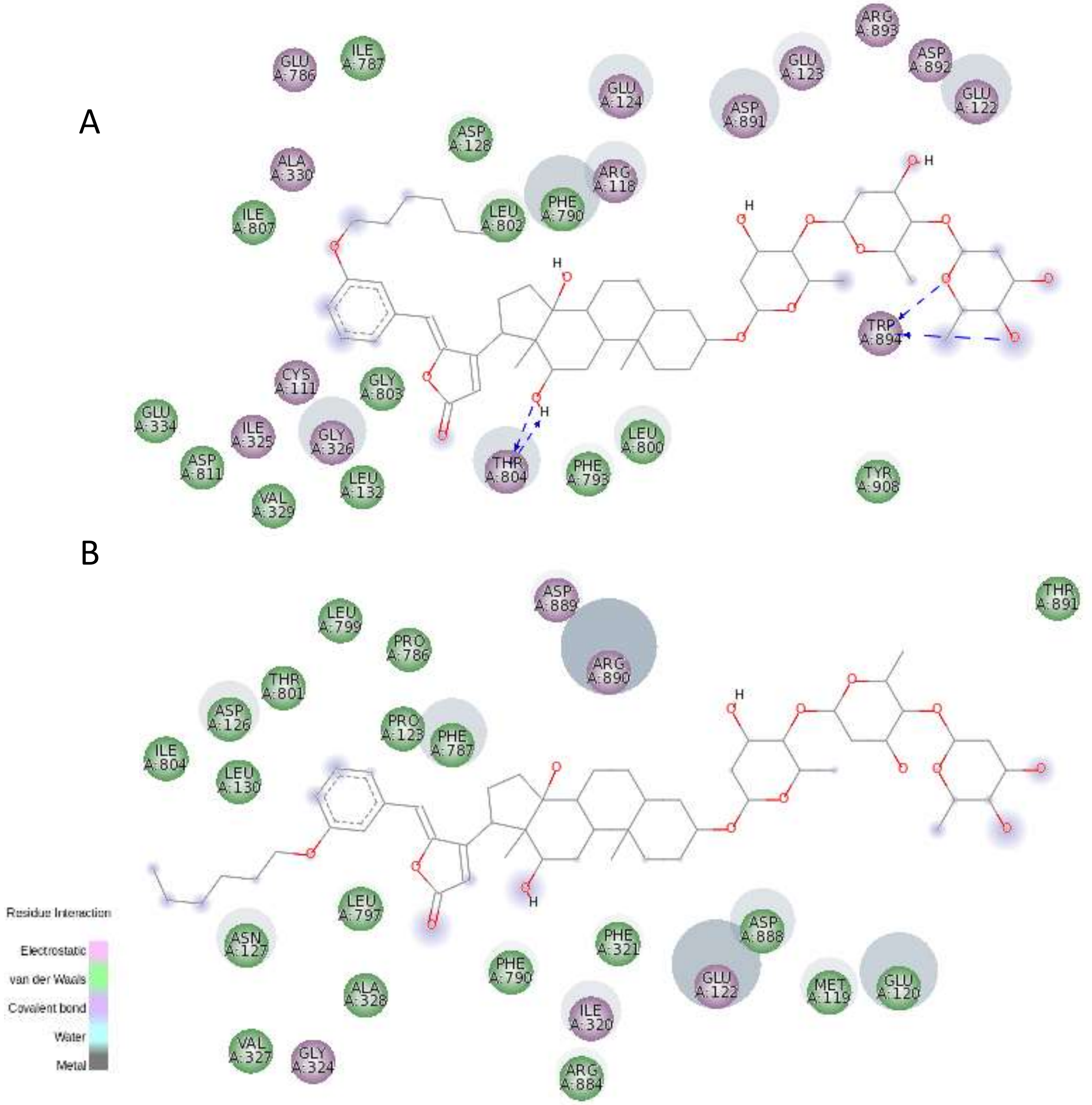
A
GLU A:786
ILE A:787
ARG A:893
ASP A:892
GLU A:123
GLU A:124
ASP A:891
GLU A:122
ASP A:128
ALA A:330
ARG A:118
ILE A:807
LEU A:802
PHE A:790
CYS A:111
GLY A:803
TRP A:894
GLU A:334
ILE A:325
GLY A:326
LEU A:800
ASP A:811
THR A:804
PHE A:793
TYR A:908
VAL A:329
LEU A:132
B
ASP A:889
THR A:891
LEU A:799
PRO A:786
ARG A:890
THR A:801
ASP A:126
PRO A:123
PHE A:787
ILE A:804
LEU A:130
LEU A:797
ASN A:127
PHE A:321
ASP A:888
PHE A:790
ALA A:328
GLU A:122
MET A:119
GLU A:120
ILE A:320
VAL A:327
GLY A:324
ARG A:884
Residue Interaction
Electrostatic
van der Waals
Covalent bond
Water
Metal

Figure S11

A

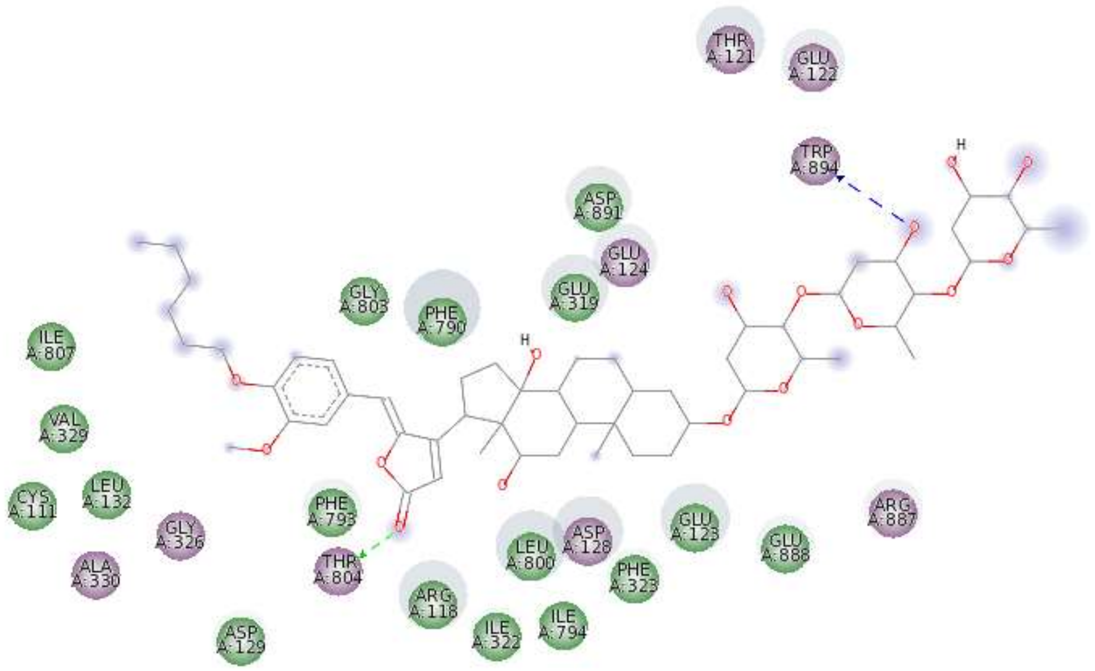

B

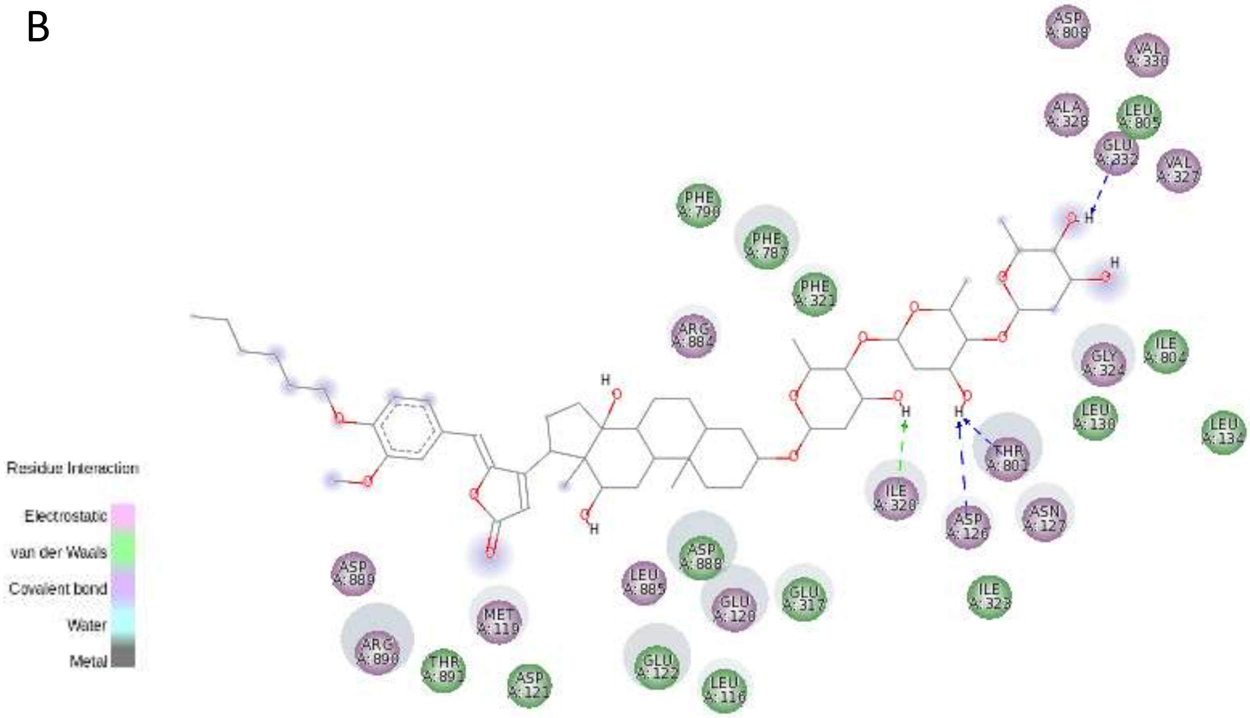

TABLES

Table S1: Estimated binding energy (kcal/mol) for CTS-NKA complexes

Compounds	α1	α2	α3
**Ouabain**	-10.3	-10.3	-11.0
**21-BD**	-12.0	-11.2	-11.6
**BD-3**	-12.1	-11.0	-10.5
**BD-8**	-11.4	-10.2	-9.4
**BD-10**	-11.4	-9.9	-9.5
**BD-13**	-10.8	-8.8	-8.9
**BD-14**	-10.9	-9.1	-9.1
**BD-15**	-9.2	-8.6	-9.2

Table S2: Total ATPase activity, residual ATPase activity and specific Na,K-ATPase activity (mean ± SD) of the membrane preparations.

Isoforms	Total ATPase activity (nmol Pi/mg protein/min)	Residual ATPase activity (upon ouabain inhibition) (nmol Pi/mg protein/min)	Specific Na,K-ATPase activity (nmol Pi/mg protein/min)
**α1β1**	43.04 ± 4.66	33.68 ± 4.19	9.36 ± 2.77
**α2β1**	46.77 ± 10.60	39.62 ± 9.62	7.15 ± 2.29
**α3β1**	53.13 ± 16.00	44.08 ± 14.20	9.05 ± 3.24
